# New evidence on the identity of the European *Helorus* species (Hymenoptera, Proctotrupoidea, Heloridae)

**DOI:** 10.3897/BDJ.12.e122523

**Published:** 2024-06-17

**Authors:** Jonathan Vogel, Jerome Sauren, Ralph S. Peters

**Affiliations:** 1 Leibniz Institute for the Analysis of Biodiversity Change, Museum Koenig Bonn, Bonn, Germany Leibniz Institute for the Analysis of Biodiversity Change, Museum Koenig Bonn Bonn Germany

**Keywords:** CO1 barcoding, integrative taxonomy, *Helorus*, species delimitation

## Abstract

**Background:**

Species of *Helorus* Latreille 1802 are rarely collected endoparasitoids of Chrysopidae larvae (Neuroptera). Previous work on the limits between the European species of this species-poor genus, based on morphology only, has left some uncertainties. Here, we approach these cases and revisit previous taxonomic decisions using freshly collected and museum material.

**New information:**

We generated the first large-scale Heloridae DNA barcode dataset, combined these with morphological data in an integrative taxonomic approach, and added information from studying all relevant type material. We found five species, *Helorusanomalipes* (Panzer, 1798), *H.coruscus* Haliday, 1857 stat. rev., *H.nigripes* Förster, 1856, *H.ruficornis* Förster, 1856, and *H.striolatus* Cameron, 1906, for which we provide an updated identification key. DNA barcode data are added to publicly available DNA barcode reference databases, for all species, except *H.nigripes*.

## Introduction

The family Heloridae Förster, 1856 (Hymenoptera, Proctotrupoidea), includes one extant genus, *Helorus* Latreille, 1802, with currently 19 valid species worldwide (Zhang et al. 2020). They are endoparasitoids of the larvae of chrysopid lacewings (Neuroptera, Chrysopidae, Chrysopinae, *Chrysopa* species) (Van Achterberg 2006). The host is killed after the wasp larva spins a cocoon and the adult wasp emerges from the host cocoon after pupating within (Townes 1977). The family is widespread and occurs in all major zoogeographic regions, but seems to be most species-rich in the Palaearctic Region (Van Achterberg 2006, Zhang et al. 2020).

In this study, we aim to contribute to our understanding of species limits in the central European species of *Helorus*. To achieve this, we gathered molecular sequence data from the DNA barcode region (a portion of the 5' end of the mitochondrial cytochrome oxidase 1 gene), as well as morphological data and combined them in an integrative taxonomic approach, also considering all relevant type specimens. For DNA barcoding, we collected fresh material from different regions of Germany, complemented by material from Belgium, and processed it following the established workflow of GBOL/GBOL III Dark Taxa (e.g. Jafari et al. (2023)). For type studies, we located, loaned and imaged type specimens from different European museums, some of which had never been examined by the authors of the previous taxonomic treatments of the genus (Pschorn-Walcher 1955, Townes 1977, Van Achterberg 2006).

So far, four species were considered valid from central Europe: *Helorusanomalipes* (Panzer, 1798), *H.ruficornis* Förster, 1856, *H.striolatus* Cameron, 1906, and the rare *H.nigripes* Förster, 1856. While *H.anomalipes* and *H.nigripes* are both easily recognised and clearly separated from all other species (Van Achterberg 2006), *H.ruficornis* Förster, 1856 and *H.striolatus* Cameron, 1906 reportedly harbour ambiguities (Townes 1977, Meyer 2001) which we specifically address in this study. First, *H.ruficornis* includes *Heloruscoruscus* Haliday, 1857, which was synonymised by Townes (1977). The somewhat dubious statements of Townes (1977), his reference to the species treatment of Pschorn-Walcher (1955), as well as the assessment of Meyer (2001) suggesting two separate species, motivated us to find further evidence speaking either for or against the conspecificity of the two names. Second, *H.striolatus* includes *Helorusmeridionalis* Pschorn-Walcher, 1955, which was also synonymised by Townes (1977). As in the first case, the treatment by Townes (1977) is not fully conclusive and the identities of these two names must be further scrutinised. Neither of these two taxonomic ambiguities were mentioned or addressed in the latest paper on European *Helorus* (Van Achterberg 2006). There are two additional documents (accessible, but not published in the sense of the International Code of Zoological Nomenclature) treating European Heloridae: Pripic-Schäper (2010), a privately published essay) and Mühlhäuser (2014), a BSc thesis at Lund University). Both indicate a separation of *H.coruscus* and *H.ruficornis*, but do not include formal taxonomic action. Neither provide any additional data on the status of *H.striolatus* and *H.meridionalis*.

The combination of the first available DNA barcode data from a considerable number of freshly-collected specimens with morphological data of voucher and type specimens allows for some new insights into the identity of the included species. New results and the DNA barcode data deposited in publicly available reference databases (bolgermany.de/ergebnisse/results; boldsystems.org) will make these few, but fascinating species accessible for future ecological and evolutionary studies, conservation, and wider recognition in science and public.

## Materials and methods

### Institutional abbreviations

NHMUIO - Naturhistorisk Museum, Oslo, Norway

NHMUK - Natural History Museum, London, United Kingdom

NHMW - Naturhistorisches Museum Wien, Vienna, Austria

NMINH - National Museum of Ireland – Natural History, Dublin, Ireland

RMNH - Nationaal Natuurhistorisch Museum, Leiden, Netherlands

SMNS - Staatliches Museum für Naturkunde Stuttgart, Germany

ZFMK - Museum Koenig Bonn, Germany

ZSM - Zoologisches Staatsmuseum München, Germany

### Specimens

We studied 49 ethanol-preserved specimens which were collected using Malaise traps, sweep netting and light traps at eight different sites in Belgium and Germany between June 2013 and August 2021. The specimens were databased with their metadata, identified to genus level, and preserved in 96% ethanol at 4°C or lower. Additional material came from museum collections, as specified in the respective material examined section.

### Preparation, imaging and pterostigma index measurements

**Figure 1.**
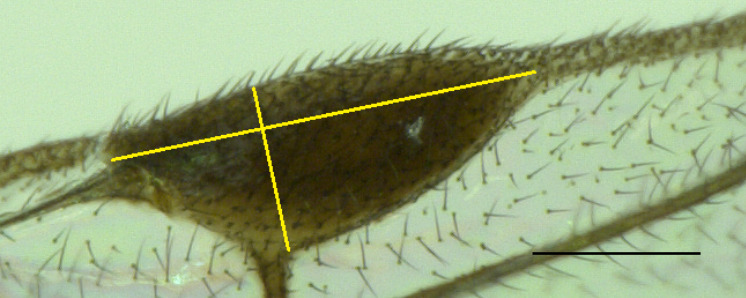
Pterostigma of fore wing of a *Helorus* specimen. The yellow lines indicate the distances we used to measure the pterostigma index (pterostigma length:width). Scale bar 200 µm.

We point-mounted (after DNA extraction, see below) the specimens and tentatively identified and sexed them according to Van Achterberg (2006). We used a Keyence VHX-2000 digital microscope to produce multi-focus images that were stacked with the built-in programme. As reference for morphological terminology, we followed Van Achterberg (2006). We measured the pterostigma indices (pterostigma length:width) from images as shown in Fig. 1 from all species, except *H.anomalipes*. In addition to the pterostigma index, we measured the petiole index (petiole length:width in dorsal view) with an eyepiece micrometer mounted on a Leica M205 C stereo microscope and the flagellomere 1 and 2 indices of selected specimens. A table with all measurements is attached in Suppl. material 1.

In all morphological descriptions, we indicate values of the primary types in parentheses in the respective species treatments.

### Molecular analysis

We extracted DNA from ethanol-preserved specimens at the Center for Molecular Biodiversity Research (ZMB) at the ZFMK in Bonn by following the procedures and protocols as described in Jafari et al. (2023). We amplified the mtDNA barcode region of the CO1 gene using the primers shown in **Table 1**. The sequencing was performed at BGI (Hong Kong). The barcodes fulfil all necessary criteria (in short: 1. Sequence length >= 500 bp, 2. bin quality = high, 3. ≤ 1% ambiguities/disagreements between the forward and reverse reads) to meet the defined GBOL gold standard (see Jafari et al. (2023) for more details). We added some outgroup sequences downloaded from BOLD (*Cratomus* sp. BOLD-ID: OPPEG1764-17, *Exallonyx* sp. BOLD-ID: BCHYM4222-14). We aligned the sequences using the built in MUSCLE alignment algorithm with a maximum of eight iterations (Edgar 2004) in GENEIOUS PRIME v.2022.1.1 (Biomatters Ltd.). The dataset can be accessed via bolgermany.de (German Barcode of Life Consortium et al. 2011) and on boldsystems.org (Ratnasingham and Hebert 2007). We list all respective BOLD-IDs of all specimens used in Suppl. material 2.

Using IQ-TREE v.2.2.2.6 (Minh et al. 2020), we reconstructed a maximum likelihood tree and used ultrafast bootstrap to calculate branch support values (Hoang et al. 2018) without further specifications. For automated species delimitation, we applied the ASAP algorithm (via https://bioinfo.mnhn.fr/abi/public/asap/, default settings, accessed 14 February 2024, Puillandre et al. (2020)). Further, we applied multirate PTP (hereafter referenced as mPTP, Kapli et al. (2017)) via a web server (https://mptp.h-its.org/#/tree, accessed 14 February 2024) to the tree file that was previously rooted with *Cratomus* sp. in FigTree v.1.4.4 (Andrew Rambaut, https://github.com/rambaut/figtree/releases/tag/v1.4.4). We used Inkscape v.1.3 (Inkscape project) to combine the final tree and the species delimitation results.

For the molecular characterisation of species, we analysed the distance matrix (Suppl. material 3) from the alignment provided in GENEIOUS to extract maximum intraspecific distances and minimum interspecific distances, stating the name of the closest species in parentheses. We generated a consensus sequence by aligning the sequences of each species separately in Geneious.

## Taxon treatments

### Helorus anomalipes

(Panzer, 1798)

BCFFBA29-44C2-5980-A4FB-44BEC6178A48

#### Materials

- **Type status:** Other material. **Occurrence:** recordedBy: Niehuis, Oliver; individualCount: 1; sex: female; disposition: in collection; associatedSequences: GBHYG1779-23; occurrenceID: ACC76843-5E42-5DA8-A2B1-5EC790FAD420; **Taxon:** family: Heloridae; genus: Helorus; specificEpithet: *anomalipes*; scientificNameAuthorship: (Panzer, 1798); **Location:** country: Germany; countryCode: DE; stateProvince: Hesse; municipality: Rheingau-Taunus; locality: Lorch am Rhein, oberhalb der Burg Nollig; verbatimElevation: 244 m; decimalLatitude: 50.0491; decimalLongitude: 7.7978; **Event:** eventID: 14; samplingProtocol: Malaise trap; eventDate: 2013-7-15/21; year: 1798; habitat: MF1; **Record Level:** institutionID: ZFMK; collectionID: ZFMK-TIS-2628158; basisOfRecord: PreservedSpecimen
- **Type status:** Other material. **Occurrence:** recordedBy: Niehuis, Oliver; individualCount: 1; sex: female; disposition: in collection; associatedSequences: upload pending; occurrenceID: BE110362-97C3-53B0-B71D-06C7C5A9FB60; **Taxon:** family: Heloridae; genus: Helorus; specificEpithet: *anomalipes*; scientificNameAuthorship: (Panzer, 1798); **Location:** country: Germany; countryCode: DE; stateProvince: Hesse; municipality: Rheingau-Taunus; locality: Lorch am Rhein, oberhalb der Burg Nollig; verbatimElevation: 261 m; decimalLatitude: 50.0498; decimalLongitude: 7.7974; **Event:** eventID: 8; samplingProtocol: Malaise trap; eventDate: 2013-6-15/23; year: 1798; habitat: MF2; **Record Level:** institutionID: ZFMK; collectionID: ZFMK-TIS-2628243; basisOfRecord: PreservedSpecimen
- **Type status:** Other material. **Occurrence:** recordedBy: Niehuis, Oliver; individualCount: 1; sex: female; disposition: in collection; associatedSequences: GBHYG2961-24; occurrenceID: B0942DA7-1795-5527-A039-FE666DF8E833; **Taxon:** family: Heloridae; genus: Helorus; specificEpithet: *anomalipes*; scientificNameAuthorship: (Panzer, 1798); **Location:** country: Germany; countryCode: DE; stateProvince: Hesse; municipality: Rheingau-Taunus; locality: Lorch am Rhein, oberhalb der Burg Nollig; verbatimElevation: 248 m; decimalLatitude: 50.0495; decimalLongitude: 7.7966; **Event:** eventID: 25; samplingProtocol: Malaise trap; eventDate: 2015-6-17/25; year: 1798; habitat: MF3; **Record Level:** institutionID: ZFMK; collectionID: ZFMK-TIS-2629475; basisOfRecord: PreservedSpecimen
- **Type status:** Other material. **Occurrence:** individualCount: 1; sex: male; disposition: in collection; associatedSequences: no DNA barcode available; occurrenceID: D5D6038B-5D65-5A3D-B411-691367CA6013; **Taxon:** family: Heloridae; genus: Helorus; specificEpithet: *anomalipes*; scientificNameAuthorship: (Panzer, 1798); **Location:** country: Germany; countryCode: DE; stateProvince: Thuringia; **Event:** year: 1798; **Record Level:** institutionID: ZFMK; collectionID: ZFMK-HYM-00039663; basisOfRecord: PreservedSpecimen

#### CO1 barcode

n = 3. Maximum intraspecific distance: 0.2%. Minimum distance to closest species (*H.striolatus*): 12.3%. Consensus sequence (625 bp): 

AATTTTAGGTTTATCAATAAGAATTATTATTCGTATAGAATTAAGTTCACCAAATTCTTTAATTAATAATGATCAAATTTATAATTCTATTGTTACATTACATGCATTTATAATAATTTTTTTTTTTATTATACCAATTACTGTTGGAGGATTTGGAAATTGATTAACTCCTATAATATTAATATCACCTGATATATCATTTCCACGATTAAATAATTTTAGATTCTGACTTTTAATTCCAAGAATTTGTTTATTAACATTTAGAAGAATTAGAGATCAAGGCCCTGGAACAGGATGAACAATTTACCCACCTTTATCTCTTAACTTAAGTCATAGAGGTAAATCTGTAGATTTAACAATTTTATCCCTACATATTGCAGGAATTTCATCAATTTTAGCATCAATTAATTTTATTACAACAATTAATAATATAAAAATTAAATCATTTTTTATAGAAAAAATTAATTTATTTATTTGATCAATATTATTAACTACTATTCTATTATTAATTTCTTTACCTGTTTTAGCTGGAGGAATTACAATAATTTTATCAGATCGAAATTTAAATTCTTCATTTTTTGACCCAAGAGGAGGAGGAGACCCAATTCTCTATCAACATTTATTT

#### Remarks

The species treatments of *H.anomalipes* in Townes (1977) and Van Achterberg (2006) are still complete and valid and are, therefore, not repeated here. All three examined and DNA barcoded specimens herein are morphologically very similar and key to *H.anomalipes* using Van Achterberg (2006). The comparatively robust and sub-basally swollen petiole in combination with the absence of pronounced coarse reticulation on head and mesosoma (cf. *Helorusnigripes*, below) is very distinctive (Fig. 3). In the molecular analysis, the sequences form a distinct cluster. In summary, this species had already been well described and diagnosed and results herein corroborate this.

### Helorus coruscus

Haliday, 1857 stat. rev.

B8090F21-F179-5997-AAC4-0001DE20D3A2

#### Materials

- **Type status:** Other material. **Occurrence:** recordedBy: GBOL III; individualCount: 1; sex: male; disposition: in collection; associatedSequences: GBHYG1780-23; occurrenceID: 3B01B70A-65B1-5175-9B46-C835398C153A; **Taxon:** family: Heloridae; genus: Helorus; specificEpithet: *coruscus*; scientificNameAuthorship: Haliday, 1857; **Location:** country: Germany; countryCode: DE; stateProvince: Hesse; municipality: Werra-Meißner-Kreis; locality: Witzenhausen, Dohrenbach, "Gut Fahrenbach" (Loc. 9); decimalLatitude: 51.3111; decimalLongitude: 9.8513; **Event:** eventID: 147; samplingProtocol: sweep net; eventDate: 2020-10-16; year: 1857; habitat: cow meadow next to beech forest with rich vegetation; **Record Level:** institutionID: ZFMK; collectionID: ZFMK-TIS-2628160; basisOfRecord: PreservedSpecimen
- **Type status:** Other material. **Occurrence:** recordedBy: Wendt, Ingo; individualCount: 1; sex: male; disposition: in collection; associatedSequences: GBHYG2835-24; occurrenceID: 3AC99442-CD26-57CC-AFBE-43D13622FDAC; **Taxon:** family: Heloridae; genus: Helorus; specificEpithet: *coruscus*; scientificNameAuthorship: Haliday, 1857; **Location:** country: Germany; countryCode: DE; stateProvince: Baden-Württemberg; municipality: Stuttgart; locality: Wilhelma, Futtergarten; decimalLatitude: 48.804; decimalLongitude: 9.2052; **Event:** eventID: 165; samplingProtocol: Malaise trap; eventDate: 2014-10-8/22; year: 1857; **Record Level:** institutionID: ZFMK; collectionID: ZFMK-TIS-2629307; basisOfRecord: PreservedSpecimen
- **Type status:** Other material. **Occurrence:** recordedBy: ZFMK et al.; individualCount: 1; sex: male; disposition: in collection; associatedSequences: GBHYG2947-24; occurrenceID: 8C9ADB82-0DA0-594A-B587-E345AA7F1AF5; **Taxon:** family: Heloridae; genus: Helorus; specificEpithet: *coruscus*; scientificNameAuthorship: Haliday, 1857; **Location:** country: Germany; countryCode: DE; stateProvince: Rhineland-Palatinate; municipality: Ahrweiler; locality: Niederzissen, Bausenberg; verbatimElevation: 321 m; decimalLatitude: 50.4647; decimalLongitude: 7.2222; **Event:** eventID: 48; samplingProtocol: Malaise trap; eventDate: 2017-7-14/20; year: 1857; habitat: MF 5.dry grassland; **Record Level:** institutionID: ZFMK; collectionID: ZFMK-TIS-2629461; basisOfRecord: PreservedSpecimen
- **Type status:** Other material. **Occurrence:** recordedBy: ZFMK et al.; individualCount: 1; sex: male; disposition: in collection; associatedSequences: GBHYG2948-24; occurrenceID: 844A6D2D-4576-545F-BE3D-5009CB56E1C5; **Taxon:** family: Heloridae; genus: Helorus; specificEpithet: *coruscus*; scientificNameAuthorship: Haliday, 1857; **Location:** country: Germany; countryCode: DE; stateProvince: Rhineland-Palatinate; municipality: Ahrweiler; locality: Niederzissen, Bausenberg; verbatimElevation: 321 m; decimalLatitude: 50.4647; decimalLongitude: 7.2222; **Event:** eventID: 48; samplingProtocol: Malaise trap; eventDate: 2017-7-14/20; year: 1857; habitat: MF 5.dry grassland; **Record Level:** institutionID: ZFMK; collectionID: ZFMK-TIS-2629462; basisOfRecord: PreservedSpecimen
- **Type status:** Other material. **Occurrence:** recordedBy: ZFMK et al.; individualCount: 1; sex: male; disposition: in collection; associatedSequences: GBHYG2949-24; occurrenceID: DC91AA40-E5CF-50A0-BF4E-E98D5E123FEA; **Taxon:** family: Heloridae; genus: Helorus; specificEpithet: *coruscus*; scientificNameAuthorship: Haliday, 1857; **Location:** country: Germany; countryCode: DE; stateProvince: Rhineland-Palatinate; municipality: Ahrweiler; locality: Niederzissen, Bausenberg; verbatimElevation: 321 m; decimalLatitude: 50.4647; decimalLongitude: 7.2222; **Event:** eventID: 48; samplingProtocol: Malaise trap; eventDate: 2017-7-14/20; year: 1857; habitat: MF 5.dry grassland; **Record Level:** institutionID: ZFMK; collectionID: ZFMK-TIS-2629463; basisOfRecord: PreservedSpecimen
- **Type status:** Other material. **Occurrence:** recordedBy: ZFMK et al.; individualCount: 1; sex: male; disposition: in collection; associatedSequences: GBHYG2950-24; occurrenceID: D173B5C7-5F72-5EC5-B7D4-BD359ECE56E8; **Taxon:** family: Heloridae; genus: Helorus; specificEpithet: *coruscus*; scientificNameAuthorship: Haliday, 1857; **Location:** country: Germany; countryCode: DE; stateProvince: Rhineland-Palatinate; municipality: Ahrweiler; locality: Niederzissen, Bausenberg; verbatimElevation: 321 m; decimalLatitude: 50.4647; decimalLongitude: 7.2222; **Event:** eventID: 48; samplingProtocol: Malaise trap; eventDate: 2017-7-14/20; year: 1857; habitat: MF 5.dry grassland; **Record Level:** institutionID: ZFMK; collectionID: ZFMK-TIS-2629464; basisOfRecord: PreservedSpecimen
- **Type status:** Other material. **Occurrence:** recordedBy: ZFMK et al.; individualCount: 1; sex: male; disposition: in collection; associatedSequences: GBHYG2953-24; occurrenceID: A8776A21-E78A-5C50-8259-EE57617A967B; **Taxon:** family: Heloridae; genus: Helorus; specificEpithet: *coruscus*; scientificNameAuthorship: Haliday, 1857; **Location:** country: Germany; countryCode: DE; stateProvince: Rhineland-Palatinate; municipality: Ahrweiler; locality: Niederzissen, Bausenberg; verbatimElevation: 321 m; decimalLatitude: 50.4647; decimalLongitude: 7.2222; **Event:** eventID: 48; samplingProtocol: Malaise trap; eventDate: 2017-7-14/20; year: 1857; habitat: MF 5.dry grassland; **Record Level:** institutionID: ZFMK; collectionID: ZFMK-TIS-2629467; basisOfRecord: PreservedSpecimen
- **Type status:** Other material. **Occurrence:** recordedBy: ZFMK et al.; individualCount: 1; sex: male; disposition: in collection; associatedSequences: GBHYG2954-24; occurrenceID: 2BF9AB75-0A59-5491-AE9D-3A10D4CBFA7E; **Taxon:** family: Heloridae; genus: Helorus; specificEpithet: *coruscus*; scientificNameAuthorship: Haliday, 1857; **Location:** country: Germany; countryCode: DE; stateProvince: Rhineland-Palatinate; municipality: Ahrweiler; locality: Niederzissen, Bausenberg; verbatimElevation: 321 m; decimalLatitude: 50.4647; decimalLongitude: 7.2222; **Event:** eventID: 48; samplingProtocol: Malaise trap; eventDate: 2017-7-14/20; year: 1857; habitat: MF 5.dry grassland; **Record Level:** institutionID: ZFMK; collectionID: ZFMK-TIS-2629468; basisOfRecord: PreservedSpecimen
- **Type status:** Other material. **Occurrence:** recordedBy: ZFMK et al.; individualCount: 1; sex: male; disposition: in collection; associatedSequences: GBHYG2955-24; occurrenceID: 42B1FBD4-4A33-55FE-A83E-B671A3977035; **Taxon:** family: Heloridae; genus: Helorus; specificEpithet: *coruscus*; scientificNameAuthorship: Haliday, 1857; **Location:** country: Germany; countryCode: DE; stateProvince: Rhineland-Palatinate; municipality: Ahrweiler; locality: Niederzissen, Bausenberg; verbatimElevation: 321 m; decimalLatitude: 50.4647; decimalLongitude: 7.2222; **Event:** eventID: 48; samplingProtocol: Malaise trap; eventDate: 2017-7-14/20; year: 1857; habitat: MF 5.dry grassland; **Record Level:** institutionID: ZFMK; collectionID: ZFMK-TIS-2629469; basisOfRecord: PreservedSpecimen
- **Type status:** Other material. **Occurrence:** recordedBy: ZFMK et al.; individualCount: 1; sex: male; disposition: in collection; associatedSequences: GBHYG2956-24; occurrenceID: 49101134-DE11-5E41-93FD-13FDE3DB7501; **Taxon:** family: Heloridae; genus: Helorus; specificEpithet: *coruscus*; scientificNameAuthorship: Haliday, 1857; **Location:** country: Germany; countryCode: DE; stateProvince: Rhineland-Palatinate; municipality: Ahrweiler; locality: Niederzissen, Bausenberg; verbatimElevation: 321 m; decimalLatitude: 50.4647; decimalLongitude: 7.2222; **Event:** eventID: 48; samplingProtocol: Malaise trap; eventDate: 2017-7-14/20; year: 1857; habitat: MF 5.dry grassland; **Record Level:** institutionID: ZFMK; collectionID: ZFMK-TIS-2629470; basisOfRecord: PreservedSpecimen
- **Type status:** Other material. **Occurrence:** recordedBy: ZFMK et al.; individualCount: 1; sex: female; disposition: in collection; associatedSequences: GBHYG2957-24; occurrenceID: F145997E-4523-58DC-8B87-C81CE62B8BE5; **Taxon:** family: Heloridae; genus: Helorus; specificEpithet: *coruscus*; scientificNameAuthorship: Haliday, 1857; **Location:** country: Germany; countryCode: DE; stateProvince: Rhineland-Palatinate; municipality: Ahrweiler; locality: Niederzissen, Bausenberg; verbatimElevation: 321 m; decimalLatitude: 50.4647; decimalLongitude: 7.2222; **Event:** eventID: 48; samplingProtocol: Malaise trap; eventDate: 2017-7-14/20; year: 1857; habitat: MF 5.dry grassland; **Record Level:** institutionID: ZFMK; collectionID: ZFMK-TIS-2629471; basisOfRecord: PreservedSpecimen
- **Type status:** Other material. **Occurrence:** recordedBy: ZFMK et al.; individualCount: 1; sex: male; disposition: in collection; associatedSequences: GBHYG2958-24; occurrenceID: 3C208580-4659-53FA-8960-52C908882D5E; **Taxon:** family: Heloridae; genus: Helorus; specificEpithet: *coruscus*; scientificNameAuthorship: Haliday, 1857; **Location:** country: Germany; countryCode: DE; stateProvince: Rhineland-Palatinate; municipality: Ahrweiler; locality: Niederzissen, Bausenberg; verbatimElevation: 321 m; decimalLatitude: 50.4647; decimalLongitude: 7.2222; **Event:** eventID: 48; samplingProtocol: Malaise trap; eventDate: 2017-7-14/20; year: 1857; habitat: MF 5.dry grassland; **Record Level:** institutionID: ZFMK; collectionID: ZFMK-TIS-2629472; basisOfRecord: PreservedSpecimen
- **Type status:** Other material. **Occurrence:** recordedBy: ZFMK et al.; individualCount: 1; sex: male; disposition: in collection; associatedSequences: GBHYG2959-24; occurrenceID: 677B3192-F88F-573B-962B-7F6020FC4027; **Taxon:** family: Heloridae; genus: Helorus; specificEpithet: *coruscus*; scientificNameAuthorship: Haliday, 1857; **Location:** country: Germany; countryCode: DE; stateProvince: Rhineland-Palatinate; municipality: Ahrweiler; locality: Niederzissen, Bausenberg; verbatimElevation: 321 m; decimalLatitude: 50.4647; decimalLongitude: 7.2222; **Event:** eventID: 48; samplingProtocol: Malaise trap; eventDate: 2017-7-14/20; year: 1857; habitat: MF 5.dry grassland; **Record Level:** institutionID: ZFMK; collectionID: ZFMK-TIS-2629473; basisOfRecord: PreservedSpecimen
- **Type status:** Other material. **Occurrence:** recordedBy: ZFMK et al.; individualCount: 1; sex: male; disposition: in collection; associatedSequences: GBHYG2960-24; occurrenceID: A59B26A1-84B7-5C62-9119-6CEFD5ED7BE8; **Taxon:** family: Heloridae; genus: Helorus; specificEpithet: *coruscus*; scientificNameAuthorship: Haliday, 1857; **Location:** country: Germany; countryCode: DE; stateProvince: Rhineland-Palatinate; municipality: Ahrweiler; locality: Niederzissen, Bausenberg; verbatimElevation: 321 m; decimalLatitude: 50.4647; decimalLongitude: 7.2222; **Event:** eventID: 48; samplingProtocol: Malaise trap; eventDate: 2017-7-14/20; year: 1857; habitat: MF 5.dry grassland; **Record Level:** institutionID: ZFMK; collectionID: ZFMK-TIS-2629474; basisOfRecord: PreservedSpecimen
- **Type status:** Other material. **Occurrence:** recordedBy: Gilgenbach, Carolin; individualCount: 1; sex: male; disposition: in collection; associatedSequences: GBHYG3706-24; occurrenceID: 1E9CD1E8-C093-540C-8A61-8D033E2B6987; **Taxon:** family: Heloridae; genus: Helorus; specificEpithet: *coruscus*; scientificNameAuthorship: Haliday, 1857; **Location:** country: Germany; countryCode: DE; stateProvince: Rhineland-Palatinate; municipality: Alzey-Worms; locality: Wine fields north of Monsheim; verbatimElevation: 145 m; decimalLatitude: 49.6406; decimalLongitude: 8.213699999999999; **Event:** eventID: 1101; samplingProtocol: Malaise trap; eventDate: 2021-8-5/24; year: 1857; habitat: shrub islands between wine fields, mostly poplars; **Record Level:** institutionID: ZFMK; collectionID: ZFMK-TIS-2632746; basisOfRecord: PreservedSpecimen
- **Type status:** Other material. **Occurrence:** recordedBy: Jan Soors; individualCount: 1; sex: female; disposition: in collection; associatedSequences: GBHYG1848-23; occurrenceID: 349EA166-0AB7-5A08-96B9-68813736DF28; **Taxon:** family: Heloridae; genus: Helorus; specificEpithet: *coruscus*; scientificNameAuthorship: Haliday, 1857; **Location:** country: Belgium; countryCode: BE; stateProvince: Antwerpen; municipality: Mechelen; decimalLatitude: 51.032; decimalLongitude: 4.488; **Event:** eventID: 1361; samplingProtocol: light trap; eventDate: 2017-7-7/7; year: 1857; habitat: Private garden; **Record Level:** institutionID: ZFMK; collectionID: ZFMK-TIS-2635112; basisOfRecord: PreservedSpecimen
- **Type status:** Other material. **Occurrence:** recordedBy: Endrestöl, Anders; individualCount: 1; sex: female; disposition: in collection; associatedSequences: no DNA barcode available; occurrenceID: C561CC85-E601-5B8A-B91B-4D9C1B8D447B; **Taxon:** family: Heloridae; genus: Helorus; specificEpithet: *coruscus*; scientificNameAuthorship: Haliday, 1857; **Location:** country: Norway; countryCode: NO; stateProvince: Oslo; municipality: Oslo; locality: N Bleikøya; verbatimElevation: 10 m; decimalLatitude: 59.88916; decimalLongitude: 10.74232; **Event:** samplingProtocol: Malaise trap; eventDate: 2008-7/8-15/29; year: 1857; habitat: forest edge/shore; **Record Level:** institutionID: NHMUIO; collectionID: http://purl.org/nhmuio/id/ea2b6743-af51-409e-bf52-e1b5213c1154; basisOfRecord: PreservedSpecimen
- **Type status:** Other material. **Occurrence:** recordedBy: Gauss; individualCount: 1; sex: female; disposition: in collection; associatedSequences: no DNA barcode available; occurrenceID: DF1B1294-57D1-55AE-89C1-E8634EA016DD; **Taxon:** family: Heloridae; genus: Helorus; specificEpithet: *coruscus*; scientificNameAuthorship: Haliday, 1857; **Location:** country: Germany; countryCode: DE; locality: Burg Birkenhof; **Event:** eventDate: 1983-10-13; year: 1857; **Record Level:** institutionID: ZFMK; collectionID: ZFMK-HYM-00039661; basisOfRecord: PreservedSpecimen
- **Type status:** Other material. **Occurrence:** recordedBy: Rheinwald; individualCount: 1; sex: female; disposition: in collection; associatedSequences: no DNA barcode available; occurrenceID: 853C2487-9071-573B-8FF7-6B9A7E6D4B63; **Taxon:** family: Heloridae; genus: Helorus; specificEpithet: *coruscus*; scientificNameAuthorship: Haliday, 1857; **Location:** country: Germany; countryCode: DE; locality: Plieningen; **Event:** eventDate: 1962-10-16; year: 1857; **Record Level:** institutionID: ZFMK; collectionID: ZFMK-HYM-00039662; basisOfRecord: PreservedSpecimen
- **Type status:** Other material. **Occurrence:** recordedBy: Jaume-Schinkel, Santiago; individualCount: 1; sex: male; disposition: in collection; associatedSequences: no DNA barcode available; occurrenceID: 5DDB0FFD-B003-5F41-BB5D-1BB4EDA870BA; **Taxon:** family: Heloridae; genus: Helorus; specificEpithet: *coruscus*; scientificNameAuthorship: Haliday, 1857; **Location:** country: Germany; countryCode: DE; stateProvince: Rhineland-Palatinate; municipality: Ahrweiler; locality: Niederzissen, Bausenberg; verbatimElevation: 313 m; decimalLatitude: 50.4672; decimalLongitude: 7.2212; **Event:** eventID: 1396; samplingProtocol: double Malaise trap; eventDate: 2022-7-12/27; year: 1857; habitat: upper part of volcanic mountain, next to oak tree; **Record Level:** institutionID: ZFMK; collectionID: ZFMK-HYM-00039664; basisOfRecord: PreservedSpecimen
- **Type status:** Other material. **Occurrence:** recordedBy: Jaume-Schinkel, Santiago; individualCount: 1; sex: male; disposition: in collection; associatedSequences: no DNA barcode available; occurrenceID: 94091472-FC77-5565-8754-4D0190600DCC; **Taxon:** family: Heloridae; genus: Helorus; specificEpithet: *coruscus*; scientificNameAuthorship: Haliday, 1857; **Location:** country: Germany; countryCode: DE; stateProvince: Rhineland-Palatinate; municipality: Ahrweiler; locality: Niederzissen, Bausenberg; verbatimElevation: 313 m; decimalLatitude: 50.4672; decimalLongitude: 7.2212; **Event:** eventID: 1396; samplingProtocol: double Malaise trap; eventDate: 2022-7-12/27; year: 1857; habitat: upper part of volcanic mountain, next to oak tree; **Record Level:** institutionID: ZFMK; collectionID: ZFMK-HYM-00039665; basisOfRecord: PreservedSpecimen
- **Type status:** Other material. **Occurrence:** recordedBy: Jaume-Schinkel, Santiago; individualCount: 1; sex: male; disposition: in collection; associatedSequences: no DNA barcode available; occurrenceID: 4456BA83-6E82-5F92-92C7-67743FE16EED; **Taxon:** family: Heloridae; genus: Helorus; specificEpithet: *coruscus*; scientificNameAuthorship: Haliday, 1857; **Location:** country: Germany; countryCode: DE; stateProvince: Rhineland-Palatinate; municipality: Ahrweiler; locality: Niederzissen, Bausenberg; verbatimElevation: 313 m; decimalLatitude: 50.4672; decimalLongitude: 7.2212; **Event:** eventID: 1396; samplingProtocol: double Malaise trap; eventDate: 2022-7-12/27; year: 1857; habitat: upper part of volcanic mountain, next to oak tree; **Record Level:** institutionID: ZFMK; collectionID: ZFMK-HYM-00039666; basisOfRecord: PreservedSpecimen
- **Type status:** Holotype. **Occurrence:** individualCount: 1; sex: male; disposition: in collection; associatedSequences: no DNA barcode available; occurrenceID: 9517A59B-323B-55F0-996C-D93D9618375B; **Taxon:** family: Heloridae; genus: Helorus; specificEpithet: *coruscus*; scientificNameAuthorship: Haliday, 1857; **Event:** eventDate: 1882-2-20; year: 1857; **Record Level:** institutionID: NMINH; collectionID: NH: 1881.18.1; basisOfRecord: PreservedSpecimen

#### CO1 barcode

n = 16. Maximum intraspecific distance: 0.5%. Minimum distance to closest species (*H.ruficornis*): 1.8%. Consensus sequence (625 bp): 

AATTATAGGTTTATCATTAAGAATAATTATTCGAATAGAATTAAGATCACCCAATTCTCTAATTAAAAATGATCAAATTTATAATTCAATTGTTACAATACATGCCTTTATAATAATTTTTTTTTTTATTATACCAATTACTGTTGGAGGATTTGGAAATTGATTAACACCAATAATAATAATATCCCCAGATATATCATTTCCTCGAATAAATAATTTAAGATTTTGATTTTTATTACCAAGAATCTTCTTAATAATATCAGGAAGAATTATTGATCAAGGATCAGGAACAGGATGAACAATTTATCCACCATTATCATTAAATTTAGCTCATAATGGAAAATCAGTTGATTTAACTATTTTATCTCTACATATTGCTGGAATTTCATCTATTTTAGCATCAATTAATTTTATTACAACAATTTTAAATATAAAAATTAAATCATTTAATATAGAAAAAATTAATTTATTTCTTTGATCAATACTTTTAACAACAATTTTACTTCTTCTATCTTTACCAGTTTTAGCTGGAGGAATTACAATAATTTTATCAGACCGAAATTTAAACTCTTCATTTTTTGACCCAAGAGGAGGTGGTGACCCAATTCTTTTTCAACATCTATTT

#### Remarks

**Figure 2.**
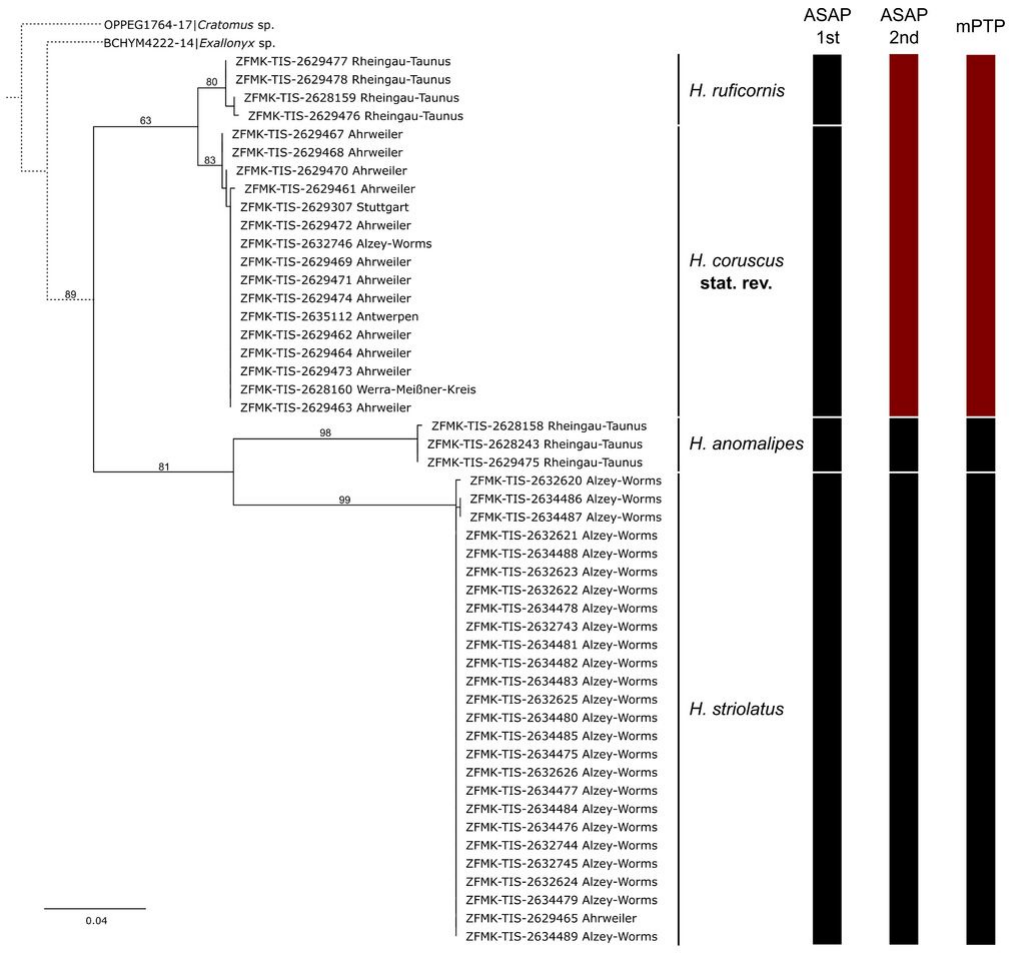
Maximum likelihood tree inferred with IQTree and the results of the molecular species delimitations. The first two ranks of ASAP have an identical score. The box colours show the results of the species delimitations that are either congruent (black) or incongruent (red) with our morphological examinations. The dotted lines connecting the outgroup are not to scale. Ultrafast-bootstrap support is shown on the branches.

**Figure 3.**
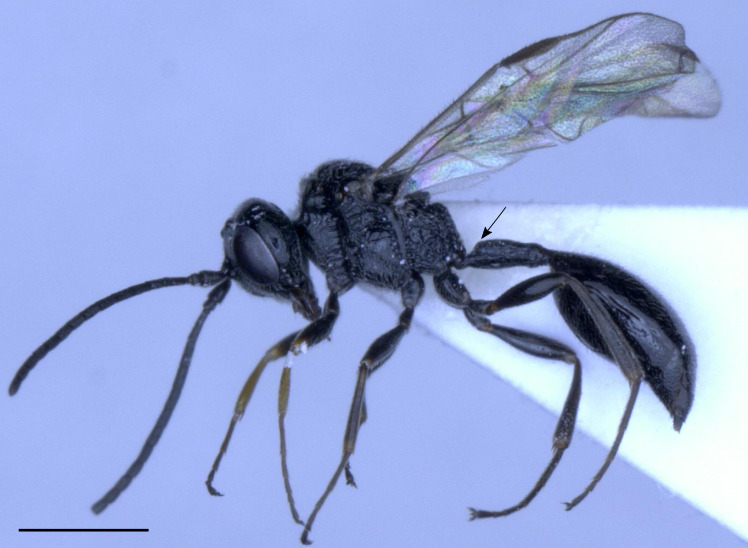
*Helorusanomalipes* (Panzer, 1798) female ZFMK-TIS-2628243, habitus lateral (arrow indicating petiole being swollen sub-basally in lateral view). Scale bar: 500 µm.

The synonymisation of *H.coruscus* under *H.ruficornis* was proposed by Townes (1977) without examining the type of *H.coruscus*. He based his action on Pschorn-Walcher (1955) who had not personally seen the type of *H.coruscus* either (spelled *H.corruscus* (sic) by Pschorn-Walcher (1955)), but for his part, based his taxonomic assessments on “Dr. Nixon, London”. This is G.E.J. Nixon (1905-1987), an esteemed specialist on various groups of parasitoid Hymenoptera. Nixon had reported to Pschorn-Walcher that he had compared some of the material from the “material examined” in Pschorn-Walcher (1955) (without further indication of which specimens in particular) with the type of *H.coruscus* in Dublin. Based on this comparison and Nixon’s statement, Pschorn-Walcher (1955) concluded that, even though they are very similar (literally “eng verwandt”), *H.ruficornis* and *H.coruscus* are two separate species. How and why Townes (1977) took these considerations to formally synonymise *H.coruscus* under *H.ruficornis* is unclear. Later, Meyer (2001) in his list of *Helorus* species from Germany also stated that he observed morphological differences between the two species in question and, ergo, listed them both, yet without formal taxonomic action. The genetic differences and the molecular species delimitation methods we applied result in ambiguity (Fig. 2). Only by a combination of the results from the analyses of DNA barcode data with the morphological examination, including the primary types of both *H.coruscus* and *H.ruficornis*, are we able to take taxonomic action and formally re-instate *H.coruscus* from synonymy.

**Figure 4.**
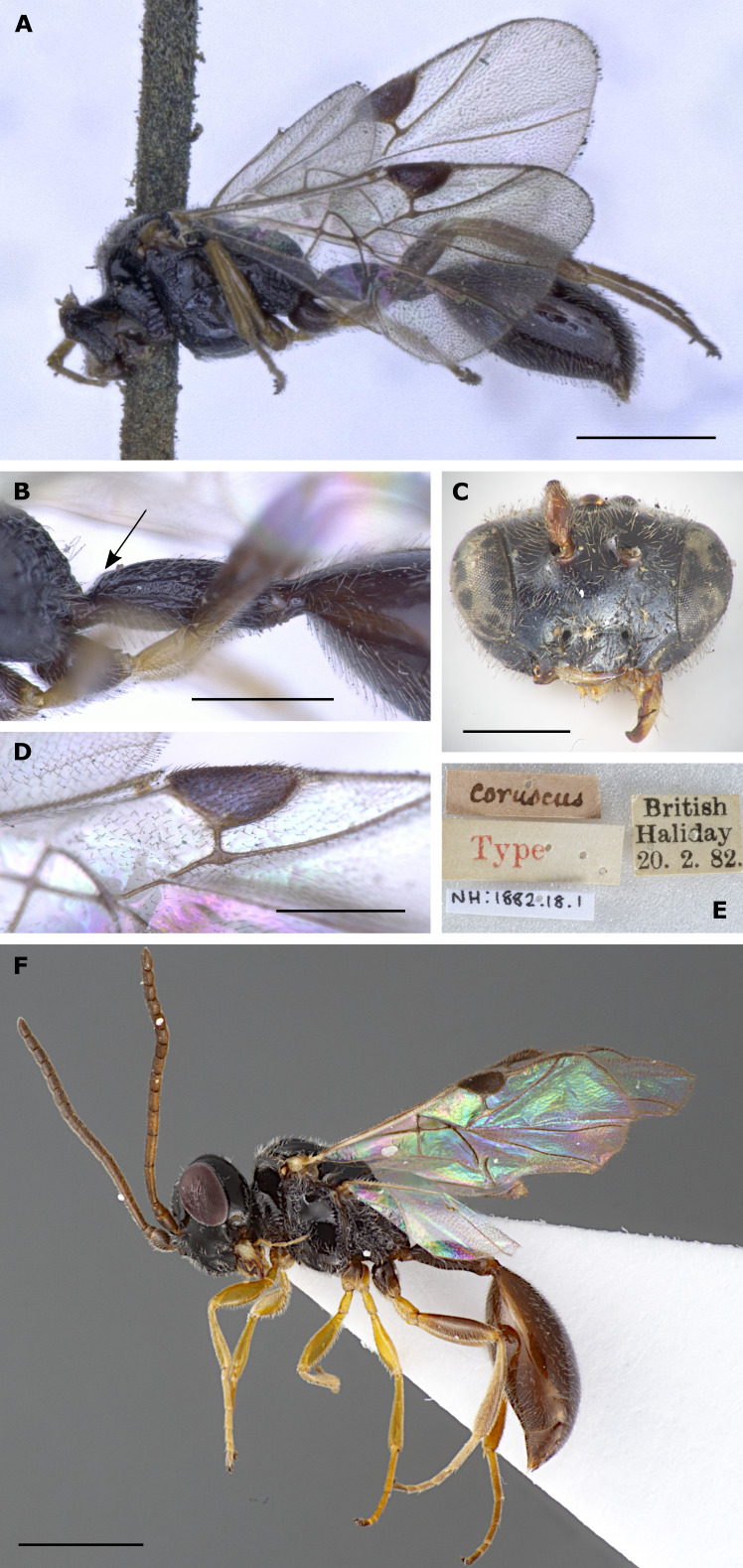
*Heloruscoruscus* Haliday, 1857. **A-E** holotype male, **A** habitus in lateral view; **B** petiole lateral (arrow indicating petiole not being distinctly swollen sub-basally in lateral view; image flipped vertically); **C** head frontal; **D** pterostigma; **E** labels; **F** female ZFMK-TIS-2629471, habitus lateral. Scale bar: **A & F** 1000 µm, **B-D** 500 µm.

**Figure 5.**
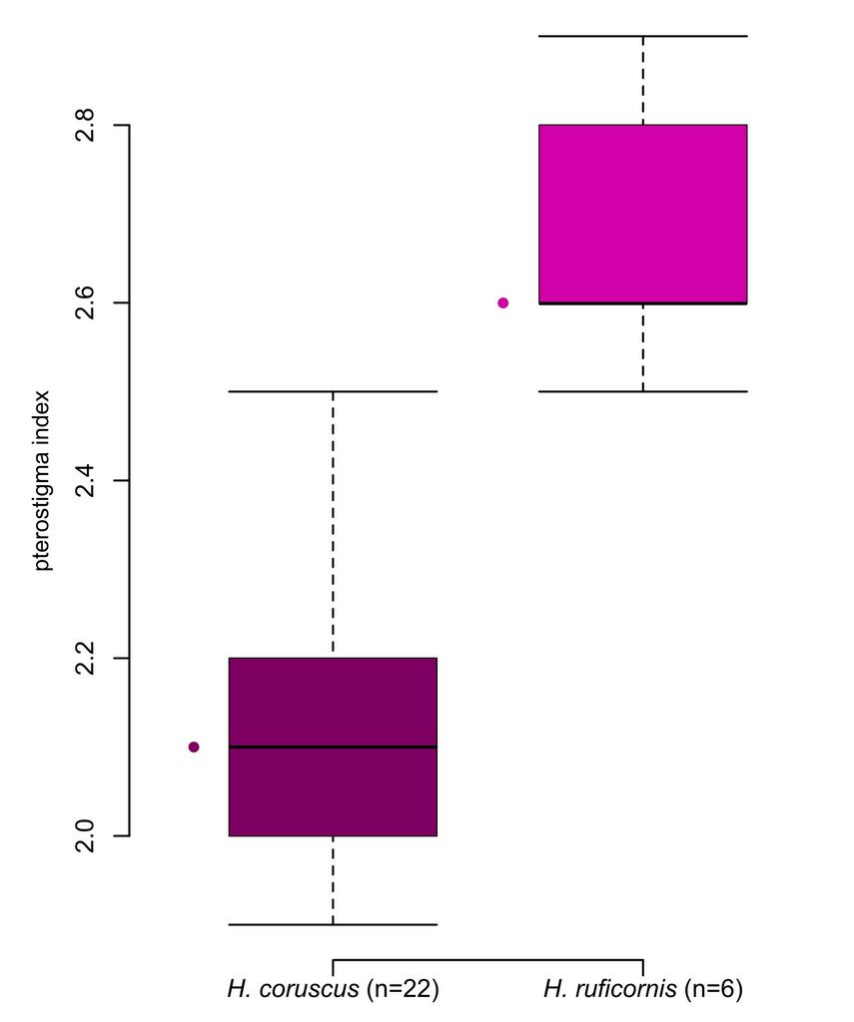
Pterostigma index values in *H.coruscus* stat. rev. and *H.ruficornis*. The values of the primary types are indicated with dots next to the boxes. Graphs were created by using R, while the data points of the types were added using Inkscape.

The two species are not easy to diagnose. The specimens examined here match the characters given by Pschorn-Walcher (1955) and Pschorn-Walcher (1971) only in part. Pschorn-Walcher (1955) does not provide a formal diagnosis of *H.coruscus*, but complements his additions to the description with characters to differentiate *H.coruscus* from *H.ruficornis* in his key to the species. Most importantly, he gives differences in the pterostigma index that we can corroborate. The pterostigma of *H.coruscus* is more robust, index 1.9–2.5 (holotype 2.1) (n = 22) (with only two specimens with a pterostigma index > 2.3) (Fig. 4D), compared to the more elongated pterostigma of *H.ruficornis* (index 2.5–2.9 (holotype 2.6) (n = 6) (Fig. 5 and Fig. 7C). The shape is comparable with the drawings of Pschorn-Walcher (1955); the range is different, though, with Pschorn-Walcher (1955) giving a pterostigma index of 2.0–2.3 for *H.coruscus* and 2.3–2.6 for *H.ruficornis*, i.e. some of his *H.ruficornis* would fall into the range of our *H.coruscus*. Additionally, Meyer (1969) gave some pterostigma values for both species, based on (a handful of) specimens from Germany, i.e. 2.1 for *H.corruscus* (sic) and 2.4 for *H.ruficornis*. Due to the low number of examined specimens, these data are of limited value, but show a more elongated pterostigma for *H.ruficornis* as well. The data provided by Mühlhäuser (2014), B.Sc. thesis) also show the same general difference; pterostigma index for *H.coruscus* (treated as a variation of *H.ruficornis*) of 2.3, for *H.ruficornis* 2.8, based on numerous specimens from Sweden. For completeness, Van Achterberg (2006), who did not differentiate between *H.coruscus* and *H.ruficornis*, gave pterostigma index values for *H.ruficornis* as 2.1–2.5 and thereby reports the combined ranges of the two species. Note that the way in which different authors measured the index may differ slightly, which might explain, for example, the (minor) difference between our index ranges and those given by Pschorn-Walcher (1955). The general conclusion that *H.coruscus* has a more robust and *H.ruficornis* has a more elongate pterostigma remains valid. In addition to the pterostigma index, the following characters were used by Pschorn-Walcher (1955) to differentiate between *H.coruscus* and *H.ruficornis*. First, the length:width ratio of flagellomeres 1 and 2 (called “Index I” and “Index II” by Pschorn-Walcher (1955)) shows longer/more slender antennal segments in *H.ruficornis* than in *H.coruscus* in both sexes. The specimens examined here exhibited overlapping values, with the exception of Index I (length:width of flagellomere 1) in females (4.0–4.2 in *H.coruscus* vs. 4.5–4.6 in *H.ruficornis* (holotype 4.6)). The sample size, however, of n = 2 per species (Suppl. material 1) is very small. Second, antennal colour is slightly different, with *H.ruficornis* having a touch of red in both sexes, hence its name and *H.coruscus* having yellow or brown antennae (Pschorn-Walcher 1955). Unfortunately, the holotype of *H.coruscus* lacks its head, including the antennae, but the fresh specimens we assigned to either species do not show any notable differences in antenna colour. Third, according to Pschorn-Walcher (1955), the hypopygium should be distinctly punctate in *H.coruscus* and less so in *H.ruficornis*. In our specimens and the respective types, we see no such distinct differences, i.e. all hypopygia are fairly distinctly punctate, with the exception of a specimen of *H.ruficornis* from the Förster collection (NHMW) that is also mentioned by Pschorn-Walcher (1955). It carries a label “type” and was collected on 11/09/1860 (see below for a statement on the lectotype designation in *H.ruficornis*). This specimen is suspiciously dull and smooth/non-punctate in the area anteriorly to the hypopygium and the hypopygium is obscured; we think this an artefact. Van Achterberg (2006) also uses hypopygium sculpture in his key, stating that, in *H.ruficornis* (including *H.coruscus*), the female hypopygium is smooth. We cannot confirm this statement (see above). Fourth, Pschorn-Walcher (1955) gives petiole index values (shorter in *H.coruscus* and longer in *H.ruficornis*, though overlapping, petiole index = length:maximum width (measured dorsally)) which we cannot corroborate, based on our fresh specimens and the types (2.3–3.1 (3.0) (n = 18) in *H.coruscus*; 2.7–3.3 (2.7) (n = 6) in *H.ruficornis*, see also Suppl. material 1). Finally, *H.coruscus* should be usually black in colour and *H.ruficornis* dark red-brown (Pschorn-Walcher 1955). After examination of fresh material and types, we find no species-specific differences in body colour. Meyer (1969) imaged male genitalia of both species; these are obviously not from the (female) holotypes and, in addition, do not seem to differ distinctly. Therefore, we refrained from examining male genitalia ourselves. The morphological diagnostic characters we found to differentiate between *H.coruscus* and other species of *Helorus*, including *H.ruficornis*, are summarised in the identification key below.

### Helorus nigripes

Förster, 1856

CF76BC05-705B-5404-B54E-0DC10EE29083

Helorus nigripes Förster, 1856: 143
Helorus rugosus Thomson, 1858: 380. Lectotype designated by Pschorn-Walcher (1955)

#### Materials

- **Type status:** Other material. **Occurrence:** individualCount: 1; sex: male; disposition: in collection; associatedSequences: no DNA barcode available; occurrenceID: B89735F0-6010-5EE8-A6D3-5208B97080BB; **Taxon:** family: Heloridae; genus: Helorus; specificEpithet: *nigripes*; scientificNameAuthorship: Förster, 1856; **Location:** country: The Netherlands; countryCode: NL; stateProvince: Limburg; municipality: Maastricht; locality: Exc. St. Pietersberg, Wijngaard, langs kleine Pruisweg; **Identification:** identifiedBy: van Achterberg, Kees; dateIdentified: 2003; **Event:** eventDate: 1950-8-16; year: 1856; **Record Level:** institutionID: RMNH; collectionID: JV_Prel_0033; basisOfRecord: PreservedSpecimen
- **Type status:** Other material. **Occurrence:** individualCount: 1; sex: female; disposition: in collection; associatedSequences: no DNA barcode available; occurrenceID: C4F9CD8A-4F22-5CD5-81BD-C72346EEB366; **Taxon:** family: Heloridae; genus: Helorus; specificEpithet: *nigripes*; scientificNameAuthorship: Förster, 1856; **Location:** country: The Netherlands; countryCode: NL; stateProvince: Limburg; municipality: Maastricht; locality: Exc. St. Pietersberg, Oosthelling bij grens; **Identification:** identifiedBy: van Achterberg, Kees; dateIdentified: 2003; **Event:** eventDate: 1950-8-17; year: 1856; **Record Level:** institutionID: RMNH; collectionID: JV_Prel_0034; basisOfRecord: PreservedSpecimen

#### Remarks

**Figure 6.**
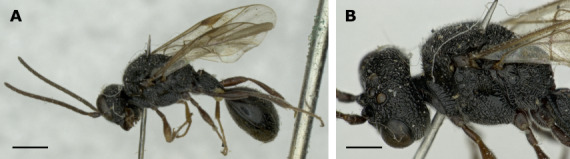
*Helorusnigripes* Förster, 1856, male (RMNH). **A** habitus lateral; **B** head and mesosoma lateral. Scale bar: **A** 1000 µm, **B** 500 µm.

**Figure 7.**
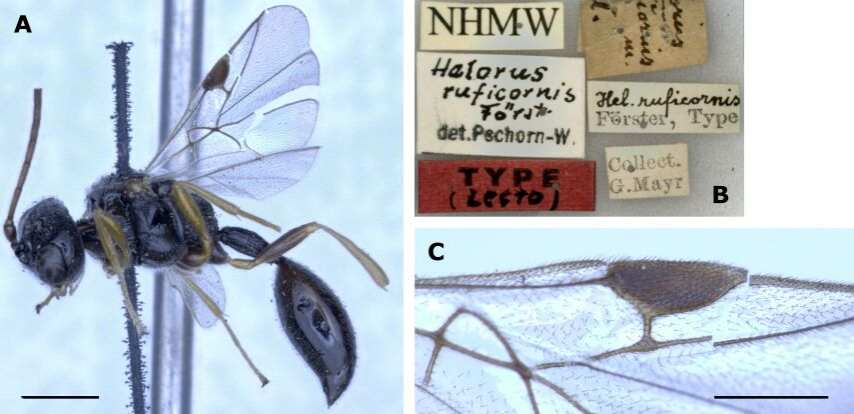
*Helorusruficornis* Förster, 1856 lectotype female (NHMW). **A** habitus lateral (image flipped vertically); **B** labels; **C** pterostigma. Scale bars: A 1000 µm, C 500 µm.

We could only study historical material of this reportedly rare species, loaned from the RMNH. The specimen shows the distinct coarse reticulation on head and mesosoma (Fig. 6B) that was already described by Förster and also mentioned, described, and imaged by subsequent authors (Pschorn-Walcher 1955, Townes 1977, Van Achterberg 2006). Note that Pschorn-Walcher (1955) wrote that the name *H.rugosus* Thomson should be preferred because Thomson’s material was, in contrast to Förster’s, still present. Pschorn-Walcher (1971) continued to use the name *H.rugosus*. Pschorn-Walcher (1955) designated a lectotype for *H.rugosus*, deposited at Stockholm Museum (SMNH). It is unclear if Townes (1977) was aware of this designation and was looking for the same specimens Pschorn-Walcher (1955) examined when he briefly reports the type as “… lost, not found in Stockholm 1975”. Despite the allegedly lost primary type of *H.nigripes*, we agree with Townes (1977) that the statement describing the unique coarse sculpture by Förster leaves no doubt about the identity of the name. The absence of the primary type of *H.nigripes* is irrelevant. Therefore, *H.nigripes* is used here, maintaining the principle of priority.

### Helorus ruficornis

Förster, 1856

742EF6B4-4F16-568E-804B-25FBC8BACC68

Helorus ruficornis Förster, 1856: 143
Helorus flavipes Kieffer, 1907: 267

#### Materials

- **Type status:** Other material. **Occurrence:** recordedBy: Niehuis, Oliver; individualCount: 1; sex: female; disposition: in collection; associatedSequences: upload pending; occurrenceID: 088D169F-1564-5BED-8633-68ED7E6BC33B; **Taxon:** family: Heloridae; genus: Helorus; specificEpithet: *ruficornis*; scientificNameAuthorship: Förster, 1856; **Location:** country: Germany; countryCode: DE; stateProvince: Hesse; municipality: Rheingau-Taunus; locality: Lorch am Rhein, oberhalb der Burg Nollig; verbatimElevation: 244 m; decimalLatitude: 50.0491; decimalLongitude: 7.7978; **Event:** eventID: 14; samplingProtocol: Malaise trap; eventDate: 2013-7-15/21; year: 1856; habitat: MF1; **Record Level:** institutionID: ZFMK; collectionID: ZFMK-TIS-2628159; basisOfRecord: PreservedSpecimen
- **Type status:** Other material. **Occurrence:** recordedBy: Niehuis, Oliver; individualCount: 1; sex: female; disposition: in collection; associatedSequences: GBHYG2962-24; occurrenceID: 98CA063E-F666-55B1-AB87-D587779D44B8; **Taxon:** family: Heloridae; genus: Helorus; specificEpithet: *ruficornis*; scientificNameAuthorship: Förster, 1856; **Location:** country: Germany; countryCode: DE; stateProvince: Hesse; municipality: Rheingau-Taunus; locality: Lorch am Rhein, oberhalb der Burg Nollig; verbatimElevation: 248 m; decimalLatitude: 50.0495; decimalLongitude: 7.7966; **Event:** eventID: 24; samplingProtocol: Malaise trap; eventDate: 2015-6/7-25/1; year: 1856; habitat: MF3; **Record Level:** institutionID: ZFMK; collectionID: ZFMK-TIS-2629476; basisOfRecord: PreservedSpecimen
- **Type status:** Other material. **Occurrence:** recordedBy: Niehuis, Oliver; individualCount: 1; sex: male; disposition: in collection; associatedSequences: GBHYG2963-24; occurrenceID: F5998079-436E-5AE3-8DCA-9879BBE8D9E5; **Taxon:** family: Heloridae; genus: Helorus; specificEpithet: *ruficornis*; scientificNameAuthorship: Förster, 1856; **Location:** country: Germany; countryCode: DE; stateProvince: Hesse; municipality: Rheingau-Taunus; locality: Lorch am Rhein, oberhalb der Burg Nollig; verbatimElevation: 244 m; decimalLatitude: 50.0491; decimalLongitude: 7.7978; **Event:** eventID: 28; samplingProtocol: Malaise trap; eventDate: 2015-6-12/17; year: 1856; habitat: MF4; **Record Level:** institutionID: ZFMK; collectionID: ZFMK-TIS-2629477; basisOfRecord: PreservedSpecimen
- **Type status:** Other material. **Occurrence:** recordedBy: Niehuis, Oliver; individualCount: 1; sex: male; disposition: in collection; associatedSequences: GBHYG2964-24; occurrenceID: 8D057C7F-36BC-5FFE-9CCA-79BE03D7DFDE; **Taxon:** family: Heloridae; genus: Helorus; specificEpithet: *ruficornis*; scientificNameAuthorship: Förster, 1856; **Location:** country: Germany; countryCode: DE; stateProvince: Hesse; municipality: Rheingau-Taunus; locality: Lorch am Rhein, oberhalb der Burg Nollig; verbatimElevation: 244 m; decimalLatitude: 50.0491; decimalLongitude: 7.7978; **Event:** eventID: 28; samplingProtocol: Malaise trap; eventDate: 2015-6-12/17; year: 1856; habitat: MF4; **Record Level:** institutionID: ZFMK; collectionID: ZFMK-TIS-2629478; basisOfRecord: PreservedSpecimen
- **Type status:** Other material. **Occurrence:** recordedBy: Doczkal, Dieter;Segerer, A.; individualCount: 1; sex: female; disposition: in collection; associatedSequences: no DNA barcode available; occurrenceID: 4ABA2156-B3B0-5396-B6B8-7D7F9D66CEB6; **Taxon:** family: Heloridae; genus: Helorus; specificEpithet: *ruficornis*; scientificNameAuthorship: Förster, 1856; **Location:** country: Germany; countryCode: DE; stateProvince: Bavaria; municipality: Regensburg; locality: Nature reserve "Fellinger Berg"; verbatimElevation: 400 m; decimalLatitude: 49.0298; decimalLongitude: 12.1567; **Event:** samplingProtocol: Malaise trap; eventDate: 2012-7-22/29; year: 1856; **Record Level:** institutionID: ZSM; collectionID: BC ZSM HYM 20589; basisOfRecord: PreservedSpecimen
- **Type status:** Lectotype. **Occurrence:** individualCount: 1; sex: female; disposition: in collection; associatedSequences: no DNA barcode available; occurrenceID: 8226F3FB-D627-55F5-B5F8-3963448A96AD; **Taxon:** family: Heloridae; genus: Helorus; specificEpithet: *ruficornis*; scientificNameAuthorship: Förster, 1856; **Event:** year: 1856; **Record Level:** institutionID: NHMW; basisOfRecord: PreservedSpecimen
- **Type status:** Paralectotype. **Occurrence:** individualCount: 1; sex: female; disposition: in collection; associatedSequences: no DNA barcode available; occurrenceID: 1988A684-B80A-5040-94BC-E7072DD4F587; **Taxon:** family: Heloridae; genus: Helorus; specificEpithet: *ruficornis*; scientificNameAuthorship: Förster, 1856; **Event:** eventDate: 1860-9-11; year: 1856; **Record Level:** institutionID: NHMW; basisOfRecord: PreservedSpecimen

#### CO1 barcode

n = 4. Maximum intraspecific distance: 0.5%. Minimum distance to closest species (*H.coruscus*): 1.8%. Consensus sequence (625 bp): 

AATTATAGGTTTATCATTAAGAATAATTATTCGAATAGAATTAAGATCACCCAATTCACTAATTAAAAATGATCAAATTTATAATTCAATTGTTACAATACATGCATTTATAATAATTTTTTTTTTTATTATACCAATTACTGTTGGAGGATTTGGAAATTGATTAACACCAATAATAATAATATCCCCAGATATATCATTTCCTCGAATAAATAATTTAAGATTTTGATTTTTAGTACCAAGAATCTTTTTAATAATATCAGGAAGAATTATTGACCAAGGATCAGGAACAGGATGAACAATTTATCCCCCATTATCATTAAATTTAGCTCATAATGGAAAATCAGTTGATTTAACTATTTTATCTCTTCATATTGCTGGAATTTCATCTATTTTAGCATCAATTAATTTTATTACAACAATTATTAATATAAAAATTAAATCATTTAATATAGAAAAAATTAATTTATTTCTTTGATCAATACTTTTAACAACAATTTTACTTCTTCTATCTTTACCAGTTTTAGCAGGAGGAATTACAATAATTTTATCAGACCGAAATTTAAACTCTTCATTTTTTGACCCAAGAGGAGGKGGKGATCCAATCCTTTTTCAACATCTATTT

#### Remarks

A full list of synonyms was recently given by Buffington and Copeland (2016) and is not repeated here. Please note, however, that Zhang et al. (2020) re-instated *H.elgoni* Risbec, 1950 as a valid species and that *H.coruscus* Haliday is re-instated herein. The synonym so far listed under *H.ruficornis*, *H.flavipes* Kieffer 1907 was considered a synonym of *H.coruscus* by Pschorn-Walcher (1955), i.e. by re-instating *H.coruscus*, it would have to be listed under that name. However, Townes (1977) saw the type material of *H.flavipes* and designated a lectotype and placed *H.flavipes* as a synonym of *H.ruficornis*. Examination of this material, which is apparently very similar to *H.coruscus* (see Pschorn-Walcher (1955)), might have given Townes (1977) the confidence to synonymise *H.coruscus* under *H.ruficornis*, without seeing the type of *H.coruscus*. We did not examine the lectotype of *H.flavipes* to decide under which name *H.flavipes* should be listed and we keep the synonymy with *H.ruficornis*, following Townes (1977).

The four specimens of *H.ruficornis* examined herein are very similar to the lectotype of *H.ruficornis* from NHMW. Note that the lectotype designation was done by Pschorn-Walcher (1955) by stating that it is a “type”, which – according to the ICZN for publications prior to 1999 – is sufficient for a valid lectotype designation. We examined a second specimen, collected on 11/09/1860, which was examined and listed as a non-type specimen by Pschorn-Walcher (1955) though it bears a hand-written label by Förster and a label “ruficornis Förster, type”. It is very similar to the *H.ruficornis* lectotype; however, the fore wings are damaged and we cannot measure its pterostigma index value. Buffington and Copeland (2016) gave some characters to differentiate *H.ruficornis* from *H.striolatus* (including pterostigma shape) and differentiation between these species is relatively easy (see also Van Achterberg (2006); and see below for remarks on the identity of *H.striolatus* and *H.meridionalis*). However, the species from which *H.ruficornis* is most difficult to distinguish is *H.coruscus*. For a discussion on the characters separating both and results from analysis of CO1 barcode data, see Fig. 2 and the treatment of *H.coruscus* above. Note that Buffington and Copeland (2016) did not contribute to the discussion of possible synonymy of *H.ruficornis* and *H.coruscus*. They also did not examine the type of *H.ruficornis*. Additionally, their values given for the petiole index (i.e. petiole length:width) of *H.ruficornis* (i.e. “4x longer than wide” in the diagnosis and “> 5-6x longer than broad” in the re-description) are not in line with the values of the *H.ruficornis* specimens examined here, including the holotype (2.7-3.3 (holotype 2.7)). This might indicate that the central European *H.ruficornis*, including the primary type, are different from the Afrotropical material examined by Buffington and Copeland (2016).

### Helorus striolatus

Cameron, 1906

306C7D19-36B3-5D20-B15C-96242E31DE42

Helorus striolatus Cameron, 1906: 98
Helorus meridionalis Pschorn-Walcher, 1955: 247

#### Materials

- **Type status:** Other material. **Occurrence:** recordedBy: ZFMK et al.; individualCount: 1; sex: male; disposition: in collection; associatedSequences: GBHYG2951-24; occurrenceID: 5A4AF6F7-E75D-5C80-8737-33AB92F3010D; **Taxon:** family: Heloridae; genus: Helorus; specificEpithet: *striolatus*; scientificNameAuthorship: Cameron, 1906; **Location:** country: Germany; countryCode: DE; stateProvince: Rhineland-Palatinate; municipality: Ahrweiler; locality: Niederzissen, Bausenberg; verbatimElevation: 321 m; decimalLatitude: 50.4647; decimalLongitude: 7.2222; **Event:** eventID: 48; samplingProtocol: Malaise trap; eventDate: 2017-7-14/20; year: 1906; habitat: MF 5.dry grassland; **Record Level:** institutionID: ZFMK; collectionID: ZFMK-TIS-2629465; basisOfRecord: PreservedSpecimen
- **Type status:** Other material. **Occurrence:** recordedBy: Gilgenbach, Carolin; individualCount: 1; sex: male; disposition: in collection; associatedSequences: GBHYG1909-24; occurrenceID: BFD5DE71-A1C6-556A-BB32-3C95D51DA696; **Taxon:** family: Heloridae; genus: Helorus; specificEpithet: *striolatus*; scientificNameAuthorship: Cameron, 1906; **Location:** country: Germany; countryCode: DE; stateProvince: Rhineland-Palatinate; municipality: Alzey-Worms; locality: Wine fields north of Monsheim; decimalLatitude: 49.6406; decimalLongitude: 8.213699999999999; **Event:** eventID: 1031; samplingProtocol: Malaise trap; eventDate: 2021-7-10/18; year: 1906; **Record Level:** institutionID: ZFMK; collectionID: ZFMK-TIS-2632620; basisOfRecord: PreservedSpecimen
- **Type status:** Other material. **Occurrence:** recordedBy: Gilgenbach, Carolin; individualCount: 1; sex: male; disposition: in collection; associatedSequences: GBHYG1910-24; occurrenceID: 641F5A38-4216-543A-979D-72DF99E43C60; **Taxon:** family: Heloridae; genus: Helorus; specificEpithet: *striolatus*; scientificNameAuthorship: Cameron, 1906; **Location:** country: Germany; countryCode: DE; stateProvince: Rhineland-Palatinate; municipality: Alzey-Worms; locality: Wine fields north of Monsheim; decimalLatitude: 49.6406; decimalLongitude: 8.213699999999999; **Event:** eventID: 1031; samplingProtocol: Malaise trap; eventDate: 2021-7-10/18; year: 1906; **Record Level:** institutionID: ZFMK; collectionID: ZFMK-TIS-2632621; basisOfRecord: PreservedSpecimen
- **Type status:** Other material. **Occurrence:** recordedBy: Gilgenbach, Carolin; individualCount: 1; sex: male; disposition: in collection; associatedSequences: GBHYG1911-24; occurrenceID: A037FC33-D657-552A-A170-62C8EE05999B; **Taxon:** family: Heloridae; genus: Helorus; specificEpithet: *striolatus*; scientificNameAuthorship: Cameron, 1906; **Location:** country: Germany; countryCode: DE; stateProvince: Rhineland-Palatinate; municipality: Alzey-Worms; locality: Wine fields north of Monsheim; decimalLatitude: 49.6406; decimalLongitude: 8.213699999999999; **Event:** eventID: 1031; samplingProtocol: Malaise trap; eventDate: 2021-7-10/18; year: 1906; **Record Level:** institutionID: ZFMK; collectionID: ZFMK-TIS-2632622; basisOfRecord: PreservedSpecimen
- **Type status:** Other material. **Occurrence:** recordedBy: Gilgenbach, Carolin; individualCount: 1; sex: male; disposition: in collection; associatedSequences: GBHYG1912-24; occurrenceID: 7DD64895-E2A1-53DB-8598-5DF2A799199A; **Taxon:** family: Heloridae; genus: Helorus; specificEpithet: *striolatus*; scientificNameAuthorship: Cameron, 1906; **Location:** country: Germany; countryCode: DE; stateProvince: Rhineland-Palatinate; municipality: Alzey-Worms; locality: Wine fields north of Monsheim; decimalLatitude: 49.6406; decimalLongitude: 8.213699999999999; **Event:** eventID: 1031; samplingProtocol: Malaise trap; eventDate: 2021-7-10/18; year: 1906; **Record Level:** institutionID: ZFMK; collectionID: ZFMK-TIS-2632623; basisOfRecord: PreservedSpecimen
- **Type status:** Other material. **Occurrence:** recordedBy: Gilgenbach, Carolin; individualCount: 1; disposition: in collection; associatedSequences: GBHYG1913-24; occurrenceID: 68E9F184-D33C-577E-8569-ABBFE163103A; **Taxon:** family: Heloridae; genus: Helorus; specificEpithet: *striolatus*; scientificNameAuthorship: Cameron, 1906; **Location:** country: Germany; countryCode: DE; stateProvince: Rhineland-Palatinate; municipality: Alzey-Worms; locality: Wine fields north of Monsheim; decimalLatitude: 49.6406; decimalLongitude: 8.213699999999999; **Event:** eventID: 1031; samplingProtocol: Malaise trap; eventDate: 2021-7-10/18; year: 1906; **Record Level:** institutionID: ZFMK; collectionID: ZFMK-TIS-2632624; basisOfRecord: PreservedSpecimen
- **Type status:** Other material. **Occurrence:** recordedBy: Gilgenbach, Carolin; individualCount: 1; sex: male; disposition: in collection; associatedSequences: GBHYG1914-24; occurrenceID: 1E31B840-4246-5C40-9154-929B882DAF4F; **Taxon:** family: Heloridae; genus: Helorus; specificEpithet: *striolatus*; scientificNameAuthorship: Cameron, 1906; **Location:** country: Germany; countryCode: DE; stateProvince: Rhineland-Palatinate; municipality: Alzey-Worms; locality: Wine fields north of Monsheim; decimalLatitude: 49.6406; decimalLongitude: 8.213699999999999; **Event:** eventID: 1031; samplingProtocol: Malaise trap; eventDate: 2021-7-10/18; year: 1906; **Record Level:** institutionID: ZFMK; collectionID: ZFMK-TIS-2632625; basisOfRecord: PreservedSpecimen
- **Type status:** Other material. **Occurrence:** recordedBy: Gilgenbach, Carolin; individualCount: 1; sex: male; disposition: in collection; associatedSequences: GBHYG1915-24; occurrenceID: D8DF02DD-C3FE-5AC4-AE20-265444E0CA44; **Taxon:** family: Heloridae; genus: Helorus; specificEpithet: *striolatus*; scientificNameAuthorship: Cameron, 1906; **Location:** country: Germany; countryCode: DE; stateProvince: Rhineland-Palatinate; municipality: Alzey-Worms; locality: Wine fields north of Monsheim; decimalLatitude: 49.6406; decimalLongitude: 8.213699999999999; **Event:** eventID: 1031; samplingProtocol: Malaise trap; eventDate: 2021-7-10/18; year: 1906; **Record Level:** institutionID: ZFMK; collectionID: ZFMK-TIS-2632626; basisOfRecord: PreservedSpecimen
- **Type status:** Other material. **Occurrence:** recordedBy: Gilgenbach, Carolin; individualCount: 1; sex: male; disposition: in collection; associatedSequences: GBHYG3703-24; occurrenceID: E40713D7-5EA2-5708-9C88-A327C7D5A83F; **Taxon:** family: Heloridae; genus: Helorus; specificEpithet: *striolatus*; scientificNameAuthorship: Cameron, 1906; **Location:** country: Germany; countryCode: DE; stateProvince: Rhineland-Palatinate; municipality: Alzey-Worms; locality: Wine fields north of Monsheim; decimalLatitude: 49.6406; decimalLongitude: 8.213699999999999; **Event:** eventID: 1031; samplingProtocol: Malaise trap; eventDate: 2021-7-10/18; year: 1906; **Record Level:** institutionID: ZFMK; collectionID: ZFMK-TIS-2632743; basisOfRecord: PreservedSpecimen
- **Type status:** Other material. **Occurrence:** recordedBy: Gilgenbach, Carolin; individualCount: 1; sex: male; disposition: in collection; associatedSequences: GBHYG3704-24; occurrenceID: 692E054D-DFF3-5CAB-AA42-59F031D9A96E; **Taxon:** family: Heloridae; genus: Helorus; specificEpithet: *striolatus*; scientificNameAuthorship: Cameron, 1906; **Location:** country: Germany; countryCode: DE; stateProvince: Rhineland-Palatinate; municipality: Alzey-Worms; locality: Wine fields north of Monsheim; decimalLatitude: 49.6406; decimalLongitude: 8.213699999999999; **Event:** eventID: 1031; samplingProtocol: Malaise trap; eventDate: 2021-7-10/18; year: 1906; **Record Level:** institutionID: ZFMK; collectionID: ZFMK-TIS-2632744; basisOfRecord: PreservedSpecimen
- **Type status:** Other material. **Occurrence:** recordedBy: Gilgenbach, Carolin; individualCount: 1; sex: male; disposition: in collection; associatedSequences: GBHYG3705-24; occurrenceID: 0B699834-7D7B-5ED0-84B0-BC8C55E1FBC7; **Taxon:** family: Heloridae; genus: Helorus; specificEpithet: *striolatus*; scientificNameAuthorship: Cameron, 1906; **Location:** country: Germany; countryCode: DE; stateProvince: Rhineland-Palatinate; municipality: Alzey-Worms; locality: Wine fields north of Monsheim; decimalLatitude: 49.6406; decimalLongitude: 8.213699999999999; **Event:** eventID: 1031; samplingProtocol: Malaise trap; eventDate: 2021-7-10/18; year: 1906; **Record Level:** institutionID: ZFMK; collectionID: ZFMK-TIS-2632745; basisOfRecord: PreservedSpecimen
- **Type status:** Other material. **Occurrence:** recordedBy: Gilgenbach, Carolin; individualCount: 1; sex: male; disposition: in collection; associatedSequences: GBHYG3772-24; occurrenceID: 9CF13F98-1E4E-5434-AA9A-DFFB23E79CA0; **Taxon:** family: Heloridae; genus: Helorus; specificEpithet: *striolatus*; scientificNameAuthorship: Cameron, 1906; **Location:** country: Germany; countryCode: DE; stateProvince: Rhineland-Palatinate; municipality: Alzey-Worms; locality: Wine fields north of Monsheim; verbatimElevation: 145 m; decimalLatitude: 49.6406; decimalLongitude: 8.213699999999999; **Event:** eventID: 1101; samplingProtocol: Malaise trap; eventDate: 2021-8-5/24; year: 1906; habitat: shrub islands between wine fields, mostly poplars; **Record Level:** institutionID: ZFMK; collectionID: ZFMK-TIS-2634475; basisOfRecord: PreservedSpecimen
- **Type status:** Other material. **Occurrence:** recordedBy: Gilgenbach, Carolin; individualCount: 1; sex: male; disposition: in collection; associatedSequences: GBHYG3773-24; occurrenceID: 4D539F16-01E1-5C58-99B4-A619E532611B; **Taxon:** family: Heloridae; genus: Helorus; specificEpithet: *striolatus*; scientificNameAuthorship: Cameron, 1906; **Location:** country: Germany; countryCode: DE; stateProvince: Rhineland-Palatinate; municipality: Alzey-Worms; locality: Wine fields north of Monsheim; verbatimElevation: 145 m; decimalLatitude: 49.6406; decimalLongitude: 8.213699999999999; **Event:** eventID: 1101; samplingProtocol: Malaise trap; eventDate: 2021-8-5/24; year: 1906; habitat: shrub islands between wine fields, mostly poplars; **Record Level:** institutionID: ZFMK; collectionID: ZFMK-TIS-2634476; basisOfRecord: PreservedSpecimen
- **Type status:** Other material. **Occurrence:** recordedBy: Gilgenbach, Carolin; individualCount: 1; sex: male; disposition: in collection; associatedSequences: GBHYG3774-24; occurrenceID: F2FF9D26-1387-5FCF-AE45-146DCE157A7A; **Taxon:** family: Heloridae; genus: Helorus; specificEpithet: *striolatus*; scientificNameAuthorship: Cameron, 1906; **Location:** country: Germany; countryCode: DE; stateProvince: Rhineland-Palatinate; municipality: Alzey-Worms; locality: Wine fields north of Monsheim; verbatimElevation: 145 m; decimalLatitude: 49.6406; decimalLongitude: 8.213699999999999; **Event:** eventID: 1101; samplingProtocol: Malaise trap; eventDate: 2021-8-5/24; year: 1906; habitat: shrub islands between wine fields, mostly poplars; **Record Level:** institutionID: ZFMK; collectionID: ZFMK-TIS-2634477; basisOfRecord: PreservedSpecimen
- **Type status:** Other material. **Occurrence:** recordedBy: Gilgenbach, Carolin; individualCount: 1; sex: male; disposition: in collection; associatedSequences: GBHYG3775-24; occurrenceID: C42860C8-A4C9-5C35-8852-872734B6F578; **Taxon:** family: Heloridae; genus: Helorus; specificEpithet: *striolatus*; scientificNameAuthorship: Cameron, 1906; **Location:** country: Germany; countryCode: DE; stateProvince: Rhineland-Palatinate; municipality: Alzey-Worms; locality: Wine fields north of Monsheim; verbatimElevation: 145 m; decimalLatitude: 49.6406; decimalLongitude: 8.213699999999999; **Event:** eventID: 1101; samplingProtocol: Malaise trap; eventDate: 2021-8-5/24; year: 1906; habitat: shrub islands between wine fields, mostly poplars; **Record Level:** institutionID: ZFMK; collectionID: ZFMK-TIS-2634478; basisOfRecord: PreservedSpecimen
- **Type status:** Other material. **Occurrence:** recordedBy: Gilgenbach, Carolin; individualCount: 1; sex: female; disposition: in collection; associatedSequences: GBHYG3776-24; occurrenceID: 4608B578-3270-5D7B-9844-F7BF985668DF; **Taxon:** family: Heloridae; genus: Helorus; specificEpithet: *striolatus*; scientificNameAuthorship: Cameron, 1906; **Location:** country: Germany; countryCode: DE; stateProvince: Rhineland-Palatinate; municipality: Alzey-Worms; locality: Wine fields north of Monsheim; verbatimElevation: 145 m; decimalLatitude: 49.6406; decimalLongitude: 8.213699999999999; **Event:** eventID: 1101; samplingProtocol: Malaise trap; eventDate: 2021-8-5/24; year: 1906; habitat: shrub islands between wine fields, mostly poplars; **Record Level:** institutionID: ZFMK; collectionID: ZFMK-TIS-2634479; basisOfRecord: PreservedSpecimen
- **Type status:** Other material. **Occurrence:** recordedBy: Gilgenbach, Carolin; individualCount: 1; sex: male; disposition: in collection; associatedSequences: GBHYG3777-24; occurrenceID: E5CD6E8B-6EC1-590B-8F44-764580275246; **Taxon:** family: Heloridae; genus: Helorus; specificEpithet: *striolatus*; scientificNameAuthorship: Cameron, 1906; **Location:** country: Germany; countryCode: DE; stateProvince: Rhineland-Palatinate; municipality: Alzey-Worms; locality: Wine fields north of Monsheim; verbatimElevation: 145 m; decimalLatitude: 49.6406; decimalLongitude: 8.213699999999999; **Event:** eventID: 1101; samplingProtocol: Malaise trap; eventDate: 2021-8-5/24; year: 1906; habitat: shrub islands between wine fields, mostly poplars; **Record Level:** institutionID: ZFMK; collectionID: ZFMK-TIS-2634480; basisOfRecord: PreservedSpecimen
- **Type status:** Other material. **Occurrence:** recordedBy: Gilgenbach, Carolin; individualCount: 1; sex: male; disposition: in collection; associatedSequences: GBHYG3778-24; occurrenceID: 68FBD5A8-C24B-5E1E-825C-5FD260E10D99; **Taxon:** family: Heloridae; genus: Helorus; specificEpithet: *striolatus*; scientificNameAuthorship: Cameron, 1906; **Location:** country: Germany; countryCode: DE; stateProvince: Rhineland-Palatinate; municipality: Alzey-Worms; locality: Wine fields north of Monsheim; verbatimElevation: 145 m; decimalLatitude: 49.6406; decimalLongitude: 8.213699999999999; **Event:** eventID: 1101; samplingProtocol: Malaise trap; eventDate: 2021-8-5/24; year: 1906; habitat: shrub islands between wine fields, mostly poplars; **Record Level:** institutionID: ZFMK; collectionID: ZFMK-TIS-2634481; basisOfRecord: PreservedSpecimen
- **Type status:** Other material. **Occurrence:** recordedBy: Gilgenbach, Carolin; individualCount: 1; sex: male; disposition: in collection; associatedSequences: GBHYG3779-24; occurrenceID: 7B175F94-3ACE-591B-AF14-E7ABAFE0FDDE; **Taxon:** family: Heloridae; genus: Helorus; specificEpithet: *striolatus*; scientificNameAuthorship: Cameron, 1906; **Location:** country: Germany; countryCode: DE; stateProvince: Rhineland-Palatinate; municipality: Alzey-Worms; locality: Wine fields north of Monsheim; verbatimElevation: 145 m; decimalLatitude: 49.6406; decimalLongitude: 8.213699999999999; **Event:** eventID: 1101; samplingProtocol: Malaise trap; eventDate: 2021-8-5/24; year: 1906; habitat: shrub islands between wine fields, mostly poplars; **Record Level:** institutionID: ZFMK; collectionID: ZFMK-TIS-2634482; basisOfRecord: PreservedSpecimen
- **Type status:** Other material. **Occurrence:** recordedBy: Gilgenbach, Carolin; individualCount: 1; sex: male; disposition: in collection; associatedSequences: GBHYG3780-24; occurrenceID: F7CAEDEA-2EE7-51BB-888D-1961EB30F9B9; **Taxon:** family: Heloridae; genus: Helorus; specificEpithet: *striolatus*; scientificNameAuthorship: Cameron, 1906; **Location:** country: Germany; countryCode: DE; stateProvince: Rhineland-Palatinate; municipality: Alzey-Worms; locality: Wine fields north of Monsheim; verbatimElevation: 145 m; decimalLatitude: 49.6406; decimalLongitude: 8.213699999999999; **Event:** eventID: 1101; samplingProtocol: Malaise trap; eventDate: 2021-8-5/24; year: 1906; habitat: shrub islands between wine fields, mostly poplars; **Record Level:** institutionID: ZFMK; collectionID: ZFMK-TIS-2634483; basisOfRecord: PreservedSpecimen
- **Type status:** Other material. **Occurrence:** recordedBy: Gilgenbach, Carolin; individualCount: 1; sex: male; disposition: in collection; associatedSequences: GBHYG3781-24; occurrenceID: ACB662BB-47A8-5A68-AC60-6AEF70B968FF; **Taxon:** family: Heloridae; genus: Helorus; specificEpithet: *striolatus*; scientificNameAuthorship: Cameron, 1906; **Location:** country: Germany; countryCode: DE; stateProvince: Rhineland-Palatinate; municipality: Alzey-Worms; locality: Wine fields north of Monsheim; verbatimElevation: 145 m; decimalLatitude: 49.6406; decimalLongitude: 8.213699999999999; **Event:** eventID: 1101; samplingProtocol: Malaise trap; eventDate: 2021-8-5/24; year: 1906; habitat: shrub islands between wine fields, mostly poplars; **Record Level:** institutionID: ZFMK; collectionID: ZFMK-TIS-2634484; basisOfRecord: PreservedSpecimen
- **Type status:** Other material. **Occurrence:** recordedBy: Gilgenbach, Carolin; individualCount: 1; sex: male; disposition: in collection; associatedSequences: GBHYG3782-24; occurrenceID: 556261B7-34B5-58EB-8B9F-A865B93F78AB; **Taxon:** family: Heloridae; genus: Helorus; specificEpithet: *striolatus*; scientificNameAuthorship: Cameron, 1906; **Location:** country: Germany; countryCode: DE; stateProvince: Rhineland-Palatinate; municipality: Alzey-Worms; locality: Wine fields north of Monsheim; verbatimElevation: 145 m; decimalLatitude: 49.6406; decimalLongitude: 8.213699999999999; **Event:** eventID: 1101; samplingProtocol: Malaise trap; eventDate: 2021-8-5/24; year: 1906; habitat: shrub islands between wine fields, mostly poplars; **Record Level:** institutionID: ZFMK; collectionID: ZFMK-TIS-2634485; basisOfRecord: PreservedSpecimen
- **Type status:** Other material. **Occurrence:** recordedBy: Gilgenbach, Carolin; individualCount: 1; sex: male; disposition: in collection; associatedSequences: GBHYG3783-24; occurrenceID: 4D9D628A-30B6-59BC-BCD8-BDA3992CBB72; **Taxon:** family: Heloridae; genus: Helorus; specificEpithet: *striolatus*; scientificNameAuthorship: Cameron, 1906; **Location:** country: Germany; countryCode: DE; stateProvince: Rhineland-Palatinate; municipality: Alzey-Worms; locality: Wine fields north of Monsheim; verbatimElevation: 145 m; decimalLatitude: 49.6406; decimalLongitude: 8.213699999999999; **Event:** eventID: 1101; samplingProtocol: Malaise trap; eventDate: 2021-8-5/24; year: 1906; habitat: shrub islands between wine fields, mostly poplars; **Record Level:** institutionID: ZFMK; collectionID: ZFMK-TIS-2634486; basisOfRecord: PreservedSpecimen
- **Type status:** Other material. **Occurrence:** recordedBy: Gilgenbach, Carolin; individualCount: 1; sex: male; disposition: in collection; associatedSequences: GBHYG3784-24; occurrenceID: 811C27D9-EEF9-5252-B602-231ADB61E0B6; **Taxon:** family: Heloridae; genus: Helorus; specificEpithet: *striolatus*; scientificNameAuthorship: Cameron, 1906; **Location:** country: Germany; countryCode: DE; stateProvince: Rhineland-Palatinate; municipality: Alzey-Worms; locality: Wine fields north of Monsheim; verbatimElevation: 145 m; decimalLatitude: 49.6406; decimalLongitude: 8.213699999999999; **Event:** eventID: 1101; samplingProtocol: Malaise trap; eventDate: 2021-8-5/24; year: 1906; habitat: shrub islands between wine fields, mostly poplars; **Record Level:** institutionID: ZFMK; collectionID: ZFMK-TIS-2634487; basisOfRecord: PreservedSpecimen
- **Type status:** Other material. **Occurrence:** recordedBy: Gilgenbach, Carolin; individualCount: 1; sex: male; disposition: in collection; associatedSequences: GBHYG3785-24; occurrenceID: D6D877F2-3998-5D41-ACA6-A6DD2B2D876D; **Taxon:** family: Heloridae; genus: Helorus; specificEpithet: *striolatus*; scientificNameAuthorship: Cameron, 1906; **Location:** country: Germany; countryCode: DE; stateProvince: Rhineland-Palatinate; municipality: Alzey-Worms; locality: Wine fields north of Monsheim; verbatimElevation: 145 m; decimalLatitude: 49.6406; decimalLongitude: 8.213699999999999; **Event:** eventID: 1101; samplingProtocol: Malaise trap; eventDate: 2021-8-5/24; year: 1906; habitat: shrub islands between wine fields, mostly poplars; **Record Level:** institutionID: ZFMK; collectionID: ZFMK-TIS-2634488; basisOfRecord: PreservedSpecimen
- **Type status:** Other material. **Occurrence:** recordedBy: Gilgenbach, Carolin; individualCount: 1; sex: female; disposition: in collection; associatedSequences: GBHYG3786-24; occurrenceID: 59577EB2-64ED-5053-81D7-96FAE261672F; **Taxon:** family: Heloridae; genus: Helorus; specificEpithet: *striolatus*; scientificNameAuthorship: Cameron, 1906; **Location:** country: Germany; countryCode: DE; stateProvince: Rhineland-Palatinate; municipality: Alzey-Worms; locality: Wine fields north of Monsheim; verbatimElevation: 145 m; decimalLatitude: 49.6406; decimalLongitude: 8.213699999999999; **Event:** eventID: 1101; samplingProtocol: Malaise trap; eventDate: 2021-8-5/24; year: 1906; habitat: shrub islands between wine fields, mostly poplars; **Record Level:** institutionID: ZFMK; collectionID: ZFMK-TIS-2634489; basisOfRecord: PreservedSpecimen
- **Type status:** Other material. **Occurrence:** recordedBy: Doczkal, Dieter; individualCount: 1; sex: female; disposition: in collection; associatedSequences: no DNA barcode available; occurrenceID: BE3908F8-ECE1-532B-904D-DB64F4FADB75; **Taxon:** family: Heloridae; genus: Helorus; specificEpithet: *striolatus*; scientificNameAuthorship: Cameron, 1906; **Location:** country: Germany; countryCode: DE; stateProvince: Bavaria; municipality: Unterfranken, Karsbach; locality: Hohhafter Berg, Southern slope; verbatimElevation: 242 m; decimalLatitude: 50.0305; decimalLongitude: 9.79618; **Event:** samplingProtocol: Malaise trap; eventDate: 2014-7/8-19/2; year: 1906; habitat: Blaugrashalde/Fels; **Record Level:** institutionID: ZSM; collectionID: BC ZSM HYM 23980; basisOfRecord: PreservedSpecimen
- **Type status:** Holotype. **Occurrence:** individualCount: 1; sex: male; disposition: in collection; associatedSequences: no DNA barcode available; occurrenceID: F2863BB2-6FF1-502D-ACEA-671AFD5FEF27; **Taxon:** family: Heloridae; genus: Helorus; specificEpithet: *striolatus*; scientificNameAuthorship: Cameron, 1906; **Location:** country: Pakistan; countryCode: PK; stateProvince: Balochistan; municipality: Quetta; **Event:** year: 1906; **Record Level:** institutionID: NHMUK; collectionID: ecatalogue:9460745; basisOfRecord: PreservedSpecimen
- **Type status:** Holotype. **Occurrence:** individualCount: 1; sex: female; disposition: in collection; associatedSequences: no DNA barcode available; occurrenceID: F00E61C0-E869-5080-A8B1-FE496D17F9CA; **Taxon:** family: Heloridae; genus: Helorus; specificEpithet: *meridionalis*; scientificNameAuthorship: Pschorn-Walcher, 1955; **Event:** year: 1955; **Record Level:** institutionID: NHMW; basisOfRecord: PreservedSpecimen
- **Type status:** Allotype. **Occurrence:** recordedBy: Mayr, G.; individualCount: 1; sex: male; disposition: in collection; associatedSequences: no DNA barcode available; occurrenceID: A8C2493E-0579-5729-80C9-B72774C43AA4; **Taxon:** family: Heloridae; genus: Helorus; specificEpithet: *meridionalis*; scientificNameAuthorship: Pschorn-Walcher, 1955; **Event:** year: 1955; **Record Level:** institutionID: NHMW; basisOfRecord: PreservedSpecimen
- **Type status:** Other material. **Occurrence:** recordedBy: Mayr, G.; individualCount: 1; sex: female; disposition: in collection; associatedSequences: no DNA barcode available; occurrenceID: D2493C4A-69BD-54DF-9A1A-80B7D6DFE8DB; **Taxon:** family: Heloridae; genus: Helorus; specificEpithet: *meridionalis*; scientificNameAuthorship: Pschorn-Walcher, 1955; **Event:** year: 1955; **Record Level:** institutionID: NHMW; basisOfRecord: PreservedSpecimen

#### CO1 barcode

n = 26. Maximum intraspecific distance: 0.3%. Minimum distance to closest species (*H.anomalipes*): 12.3%. Consensus sequence (625 bp): 

AATTATTGGTCTTTCAATAAGATTAATTATTCGAATAGAATTAAGTTCTCCTGGATCATTAATTAAAAATGATCAAATTTATAATTCTTTTGTTACTTTACATGCTTTCTTAATAATTTTTTTTTTTATTATACCAATTACTGTTGGAGGTTTTGGAAATTGATTAACACCAATAATATTAATAACCCCAGATATATCATTTCCACGAATAAATAATTTAAGATTTTGATTATTAATCCCTAGAATTTCTTTAATATTATTTAGAAGAATTAGAGATCAAGGTCCAGGAACAGGATGAACAATTTACCCACCTTTATCATTAAATTTAAGTCATAGAGGTAAAGCAGTTGATTTAACAATTTTATCACTTCATATTGCAGGAATTTCTTCAATTTTAGCCTCAATTAATTTCATTACTACAATTTTAAATATAAAAATTAAATCTTTCTCTATTGAAAAAATTAATTTATTTTTATGATCAATACTTTTAACTACAATTTTATTATTAATCTCTTTACCAGTATTAGCTGGAGGAATCACTATAATTTTATTTGATCGAAATATAAATTCTTCATTTTTTGATCCAAGAGGAGGAGGAGATCCAATCCTTTATCAACATCTATTT

#### Remarks

*H.striolatus* can be separated from all other species by the combination of a slender petiole, a long pterostigma, and darker legs (see Van Achterberg (2006) and the key below). In our analyses of molecular sequence data, specimens with these characters clearly form a separate species. In addition to our material, we examined the types of *H.striolatus* and *H.meridionalis* and found all specimens to be very similar. *Helorusmeridionalis* is a synonym of *H.striolatus* which has been synonymised by Townes (1977), again without examination of the type (of *H.meridionalis*; cf. the case of *H.coruscus* above). Synonymisation was solely based on re-evaluation of the description by Pschorn-Walcher (1955). Pschorn-Walcher (1955) did not include *H.striolatus* in his revision, because he had no material of this species at hand and did not examine the type. Therefore, he does not give any diagnostic characters to separate his new *H.meridionalis* from *H.striolatus*. Later, Townes (1977) examined the type of *H.striolatus* and found the description of *H.meridionalis* by Pschorn-Walcher (1955) sufficient to synonymise the two. Prpic-Schäper ((2011), privately published essay) outlines some doubts on the validity of the synonymisation of the two species, based on differences in the first two flagellomere indices and the differences in type locality. The type of *H.striolatus* was collected in Pakistan, i.e. geographically separated from, for example, the *H.meridionalis* type from Italy or the specimens examined herein from Germany. Furthermore, the types of *H.striolatus* and *H.meridionalis* are both lacking their heads, which makes an examination of the flagellomere lengths impossible.

The colouration of the legs is lighter in the *H.striolatus* type than in the *H.meridionalis* type, but these are only subtle colouration differences that are hardly useful for species delimitation. Van Achterberg (2006) also notes differences in *H.striolatus* specimens from Spain (quote “The specimens from Spain have the vertex strongly punctate and the pterostigma slightly more robust than other specimens”) which could point to the existence of a separate species, maybe *H.meridionalis*, in southern Europe. As the primary types of both species have lost their heads, the first character cannot be evaluated further. The pterostigma is, as Van Achterberg (2006) described, slightly more robust in the *H.meridionalis* type than in the *H.striolatus* (pterostigma index 2.9 *meridionalis* type, 3.4 *striolatus* type). While the pterostigma index value of the *H.meridionalis* lectotype falls into the range that we find in our specimens of *H.striolatus* (2.8-3.3), the holotype of *H.striolatus* exhibits an extreme value of 3.4.

**Figure 8.**
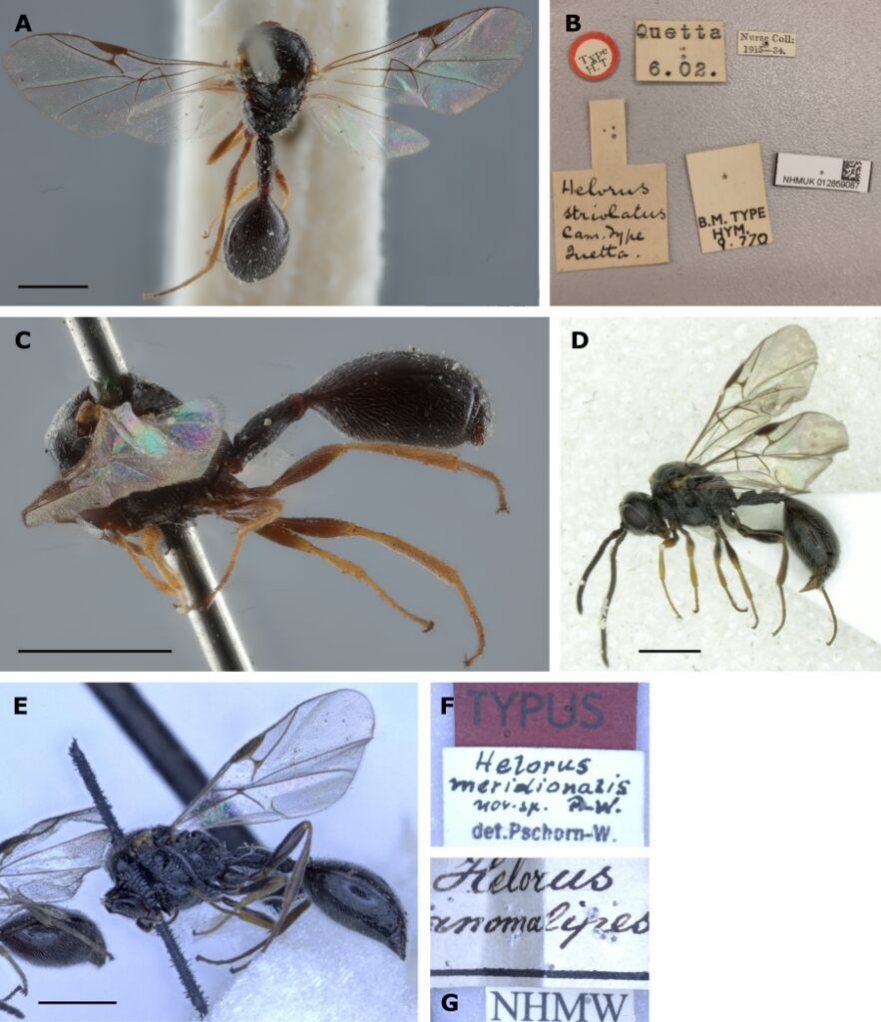
*Helorusstriolatus* Cameron, 1906 and its synonym *H.meridionalis* Pschorn-Walcher, 1955. **A-D** *Helorusstriolatus* Cameron, 1906: **A** holotype male habitus dorsal; **B** holotype labels; **C** holotype habitus lateral; **D** male ZFMK-TIS-2632620 habitus lateral. **E-F** *Helorusmeridionalis* Pschorn-Walcher, 1955: **E** holotype female habitus lateral (image flipped vertically); **F** holotype labels; **G** label of additional specimen on same pin as *H.meridionalis* holotype (also visible in **E**). Scale bars 1000 µm.

Pschorn-Walcher (1955) already points out that the lack of *H.striolatus* material he had at hand makes it impossible to decide whether it is what he calls “a good species”. Townes (1977) examined more material, but all came from the Western Palaearctic (including five specimens from Israel as the easternmost locality). Van Achterberg (2006) examined material from various countries, including Turkey, but did not aim at a taxonomic revision. From his note, that, specifically, specimens from Spain were different from other examined material (see above), we can conclude that the ones from Turkey did not differ from his central European material. The analysis of DNA barcode data herein is of limited significance because all included specimens originated from Germany; they currently do not point towards two species under the name *H.striolatus*. To investigate this further, more specimens, ideally with both morphological and molecular data, are needed from southern Europe, as well as from Pakistan and neighbouring countries and the Middle East. Izadizadeh et al. (2015) described an additional species that is most similar to *H.striolatus* from Iran. We did not examine this species and type. Currently, arguments for separation between *H.striolatus* and *H.meridionalis* and for re-instating *H.meridionalis* are weak and we keep the synonymisation and place all our specimens under the name *H.striolatus*. Please note that the pin holding the holotype female of *H.meridionalis* bears a second (male) specimen (Fig. 8E). The male allotype mentioned by Pschorn-Walcher (1955) is on a separate pin and in a very poor condition (i.e. missing head, hind wings, one fore wing, and metasoma). The male specimen that is with the holotype is indeed a *H.anomalipes* specimen. It is unknown to us who put this specimen next to the *H.meridionalis* holotype.

## Identification Keys

### Key to central European species of *Helorus* (male and female)

**Table:** 

1	Petiole robust [petiole length:width in dorsal view (= petiole index) ≤ 2.3] and swollen sub-basally in lateral view (Fig. 3, Fig. 6A)	2
–	Petiole more slender (petiole index ≥ 2.3) and not distinctly swollen sub-basally in lateral view (Fig. 4B, Fig. 7A, Fig. 8C, E)	3
2	With distinct coarse reticulation on head and mesosoma (Fig. 6B)	***H.nigripes***
–	Without distinct coarse reticulation on head and mesosoma (Fig. 3)	***H.anomalipes***
3	Fore- and mid-legs entirely yellow (except black coxae) (Fig. 4F, Fig. 7A); wings hyaline (Fig. 7C); pterostigma shorter [pterostigma length:width (= pterostigma index) 1.9-2.9]	4
–	Fore- and mid-legs darkened at coxae, trochanters and at least the base of femura (rest yellow) (Fig. 8C-E); wings slightly infuscate (not well visible in images); pterostigma longer (pterostigma index usually 3.0-3.3, rarely below 3.0)	***H.striolatus***
4	Pterostigma shorter (pterostigma index ≤ 2.5, usually ≤ 2.3, Fig. 4D), flagellomere 1 length:width in females 4.0-4.2	***H.coruscus***
–	Pterostigma longer (pterostigma index ≥ 2.5, Fig. 7C), flagellomere 1 length:width in females 4.5-4.6	***H.ruficornis***

## Analysis

All European species of the genus *Helorus* are represented in the studied material. No fresh material of *H.nigripes* was available to us for sequencing and the species is, hence, not included in the DNA barcode data and analyses. In total, 49 DNA barcode sequences were obtained and 67 specimens were examined morphologically.

The analyses of DNA barcode data imply the presence of three to four species in our material. The ASAP analysis resulted in two partitions with an identical score (1.5). Both partitions are implemented in Fig. 2 and ambigously labelled ASAP 1^st^ and ASAP 2^nd^. ASAP 1^st^ infers four, ASAP 2^nd^ three putative species. The mPTP analysis is in line with the results of ASAP 2^nd^.

We ultimately based our species delimitations for the species treatments on an integration of both automated molecular species delimitation and morphological examination. We recognise four species within the DNA barcoded material (Fig. 2).

The four clusters/species differ from each other in their DNA barcode sequence. *Heloruscoruscus* and *H.ruficornis* differ distinctly from the other two clusters/species, by a minimum of 12.5%. *Helorusanomalipes* and *H.striolatus* are clearly separated from each other by a minimum 12.3% difference. All 26 specimens of the *H.striolatus* cluster are genetically identical, although from two different locations. The difference between *H.coruscus* and *H.ruficornis* is smaller than the other interspecific differences in the dataset, with a minimum of 1.8% and a maximum of 2.4%.

For more details on the species delimitation, also including the fifth species *H.nigripes*, see the respective treatments and the identification key.

## Discussion

*Helorus* is a species-poor genus, but even these can harbour persistent taxonomic problems. Matters are further complicated by the comparative rarity of all included species which leads to small series available for taxonomic studies, both in fresh collections as well as in museum collections, even for the central European region. The addition of the first large-scale DNA barcode sequence data and the examination of the type specimens was key to our contribution and we are confident that we were able to add some clarity and enable future studies on this group of wasps. In fact, helorids have been studied neither in an evolutionary, nor an ecological or conservation context. So far, they have shared the fate of many “Dark Taxa” (sensu Hausmann et al. (2020)) because they were simply not accessible or “on the map”. We do not add any information on life history, most notably host use, so that a lot of fundamental information about the species is still missing (see Pschorn-Walcher (1955), Pschorn-Walcher (1971) and Townes (1977) for the current knowledge on host associations). We expect this study to be part of the first step towards a better understanding of these species. In this context, we also tried to make the key included here as simple as possible, in order to have users from ecology, citizen science or conservation feel invited and not repelled by excessive lists of characters that are difficult to assess by the untrained eye.

## Supplementary Material

XML Treatment for Helorus anomalipes

XML Treatment for Helorus coruscus

XML Treatment for Helorus nigripes

XML Treatment for Helorus ruficornis

XML Treatment for Helorus striolatus

B2CEDDD9-A702-5D36-B935-FE2638EF154E 10.3897/BDJ.12.e122523.suppl1 Supplementary material 1 Measurements Data type morphological Brief description Measurements of petiole index, pterostigma index and flagellomere 1 and 2 indices. File: oo_995581.xlsx https://binary.pensoft.net/file/995581 Vogel, J, Sauren, J, Peters, RS

27CB99BD-6FA7-50DE-931B-AE70096A1A38 10.3897/BDJ.12.e122523.suppl2 Supplementary material 2 Specimen and DNA barcode IDs Data type Specimen IDs, BOLD-IDs Brief description Listing all specimens used in the molecular analysis with identity, specimen ID, BOLD ID, barcode sequence length and their use as either out- or ingroup. File: oo_1024593.xlsx https://binary.pensoft.net/file/1024593 Vogel, J

E3DADF14-A5BC-5840-9F02-6D582F8B99A9 10.3897/BDJ.12.e122523.suppl3 Supplementary material 3 Distance matrix Data type molecular Brief description Distance matrix of the CO1 barcode sequences. File: oo_995584.csv https://binary.pensoft.net/file/995584 Vogel, J

## Figures and Tables

**Table 1.** Primers used for the amplification of an abbreviated barcode region for *Helorus* spp. (625bp).

Primer	Direction	Sequence length	Primer sequence	Reference
Heloridae-CV-F	forward	19	5’ TATTTGGAATATGAGCAGG 3’	Rehberger et al. (2024)
HCO2198-JJ	reverse	25	5’ AWACTTCVGGRTGVCCAAARAATCA 3’	Astrin and Stüben (2008)
